# Allopolyploid origin in *Rubus* (Rosaceae) inferred from nuclear granule-bound starch synthase I (*GBSS*I) sequences

**DOI:** 10.1186/s12870-019-1915-7

**Published:** 2019-07-10

**Authors:** Yan Wang, Qing Chen, Tao Chen, Jing Zhang, Wen He, Lin Liu, Ya Luo, Bo Sun, Yong Zhang, Hao-ru Tang, Xiao-rong Wang

**Affiliations:** 1 Institute of Pomology and Olericulture, Sichuan Agricultural University, Chengdu, China; 2 College of Horticulture, Sichuan Agricultural University, Chengdu, China; 3 Xizang Agriculture and Animal Husbandry College, Linzhi, China

**Keywords:** *Rubus*, *GBSS*I-1, Section *Idaeobatus*, Sect. *Malachobatus*, Allopolyploid origin, Hybridization, Evolution

## Abstract

**Background:**

Polyploidy and hybridization are ubiquitous in *Rubus* L., a large and taxonomically challenging genus. Chinese *Rubus* are mainly concentrated into two major sections, the diploid *Idaeobatus* and the polyploid *Malachobatus*. However, it remains unclear to be auto- or allo- polyploid origin of polyploids in *Rubus*. We investigated the homoeologs and the structure of the *GBSS*I-1 (granule-bound starch synthase I) gene in 140 *Rubus* individuals representing 102 taxa in 17 (out of the total 24) subsections of 7 (total of 12) sections at different ploidy levels.

**Results:**

Based on the gene structure and sequence divergence, we defined three gene variants, *GBSS*I-1a, *GBSS*I-1b, and *GBSS*I-1c. When compared with *GBSS*I-1a, both *GBSS*I-1b and *GBSS*I-1c have a shorter fourth intron, and *GBSS*I-1c had an additional deletion in the fifth intron. For diploids, either *GBSS*I-1a or *GBSS*I-1b was detected in 56 taxa consisting of 82 individuals from sect. *Idaeobatus*, while both alleles existed in *R. pentagonus* and *R. peltatus*. Both homoeologs *GBSS*I-1a and *GBSS*I-1b were identified in 39 taxa (48 individuals) of *Malachobatus* polyploids. They were also observed in two sect. *Dalibardastrum* taxa, in one sect. *Chamaebatus* taxon, and in three taxa from sect. *Cylactis*. Interestingly, all three homoeologs were observed in the three tetraploid taxa. Phylogenetic trees and networks suggested two clades (I and II), corresponding to *GBSS*I-1a, and *GBSS*I-1b/1c sequences, respectively. *GBSS*I-1 homoeologs from the same polyploid individual were resolved in different well-supported clades, and some of these homoelogs were more closely related to homoelogs in other species than they were to each other. This implied that the homoeologs of these polyploids were donated by different ancestral taxa, indicating their allopolyploid origin. Two kinds of diploids hybridized to form most allotetraploid species. The early-divergent diploid species with *GBSS*I-1a or -1b emerged before polyploid formation in the evolutionary history of *Rubus*.

**Conclusion:**

This study provided new insights into allopolyploid origin and evolution from diploid to polyploid within the genus *Rubus* at the molecular phylogenetic level, consistent with the taxonomic treatment by Yü et al. and Lu.

**Electronic supplementary material:**

The online version of this article (10.1186/s12870-019-1915-7) contains supplementary material, which is available to authorized users.

## Background

The genus *Rubus* L. belongs to the subfamily Rosoideae of the family Rosaceae, with 750–1000 species distributed worldwide except Antarctica [1–3]. Focke [1–3] established the widely adopted *Rubus* taxonomy that contained 12 subgenera, with the three largest subgenera of *Idaeobatus*, *Malachobatus*, and *Rubus* (Additional file 1). The number of *Rubus* species in China accounts for 97% of the total in Asia. More than 200 species have been recorded in China, of which 139 species are indigenous [4]. Basing upon the evolutionary tendency of morphological features, chromosome numbers of certain species and the distribution patterns of species, taxonomists in China [4–6] proposed a new systematic arrangement of Chinese *Rubus*, with eight sections (Additional file 1). The two taxonomic systems are concordant in the classification of most species, while the arrangement of sections is presented in a reverse order to those of Focke’s system (Additional file 1). Most species are assigned into two major sections, *Idaeobatus* and *Malachobatus*, including 11 and 13 subsections, respectively [5]. Section *Idaeobatus* is characterized by its shrub habit armed with sharp prickles, aciculae or setae, leaves pinnately compound or simple, stipules attached to the petioles, flowers hermaphroditic and often in terminal or axillary inflorescences, very rarely solitary, and drupelets separating from the receptacles [5, 6]. In contrast, members of sect. *Malachobatus* are usually woody with prickles, simple-leaved, stipules free, flowers bisexual and in cymose panicles, subracemes, and drupelets adhering to receptacles [5, 6].

The evolutionary history of *Rubus* species inferred from different analyses has been argued for a long time. Based on morphological and chromosomal data, Lu [4] suggested that evolution in *Rubus* proceeded from woody to herbaceous plants, and from species with compound leaves to simple leaves. This proposal was consistent with the view of Kalkman [7]. However, ITS data conflicted with these hypotheses: primarily semi-herbaceous, simple-leaved species occupied early-diverging positions in the trees [8].

Polyploidy and hybridization are common in *Rubus* [9]. Species of sects. *Idaeobatus* are predominantly diploids (2*n* = 2*x* = 14), while sects. *Malachobatus*, *Dalibardastrum*, and *Chamaemorus* are exclusively polyploids (2*n* = 4*x*, 6*x*, 8*x*, 14*x* = 28, 42, 56, 84) [9–11]. In addition, interspecific hybridization and facultative apomixis play an important role in sect. *Rubus*, which blurred species boundaries [9]. Based on chromosomal karyotype, meiotic pairing and fluorescence in situ hybridization (FISH) analyses, several polyploids from sect. *Malachobatus* have been demonstrated to be of allopolyploid origin [12, 13]. Hybridization in *Rubus* occurs not only between closely related species from the same section [14–21], but also between species from different sections [22, 23]. Soltis & Soltis [24] proposed that, allopolyploid formation via interspecific hybridization and subsequent genome doubling has become an important mode of speciation in higher plants. Therefore, based on the assumption and our previous studies [12, 13, 25], we speculated the majority of polyploids being of allopolyploid origin. It is needed to be further elucidated by powerful evidence.

To reconstruct the evolutionary history of plant polyploid species using molecular data, it is necessary to deal with the presence and the evolutionary fate of multiple gene copies resulting from paralogs and orthologs [26]. Identification of homoeologs in polyploids is crucial for reliable phylogeny reconstruction, and also informative for identifying parental lineages and inferring auto- or allo- formation of polyploids [27]. Low-copy nuclear genes that succeeded in other Rosaceae are potentially ideal nuclear markers for phylogenetic analysis of *Rubus* complex. The *GBSS*I gene, coding for granule-bound starch synthase I, is single copy in most diploid angiosperms [28]. The entire gene consists of 13 translated exons and 12 introns. Phylogenetic studies have shown that *GBSS*I exons and introns are useful in resolving relationships among closely related genera and species [26], especially in detecting ancient hybridization events of polyploids [29, 30]. In *Rubus* and most Rosaceae, the *GBSS*I gene is represented by two paralogous loci, *GBSS*I-1 and *GBSS*I-2, which can be differentiated by specific indels [29, 31]. Partial *GBSS*I-2 sequences, as a single copy gene, have provided high phylogenetic resolution within *Rubus* [25]. Additionally, two different alleles of *GBSS*I-1 were detected in octoploid *R. chamaemorus*, inferring it to be an ancient allopolyploid that resulted from multiple hybridization events [30]. It is believed that *GBSS*I-1 gene is extremely helpful to reveal the origin and evolution for *Rubus* polyploids.

In this study, we explored the utility of *GBSS*I-1 to elucidate the evolutionary history of genus *Rubus* and particularly the auto- or allo- polyploid origin of the polyploids. Our objectives were (i) to investigate the number of *GBSS*I-1 variants within *Rubus* at different ploidy levels, (ii) to analyze the gene structure and conduct homoeolog identification, and (iii) to provide new insights into the polyploid origin and evolutionary history within *Rubus* by reconstructing the phylogeny.

## Results

### Gene variants and orthology identification of *GBSS*I-1 within *Rubus*

**Fig. 1:**
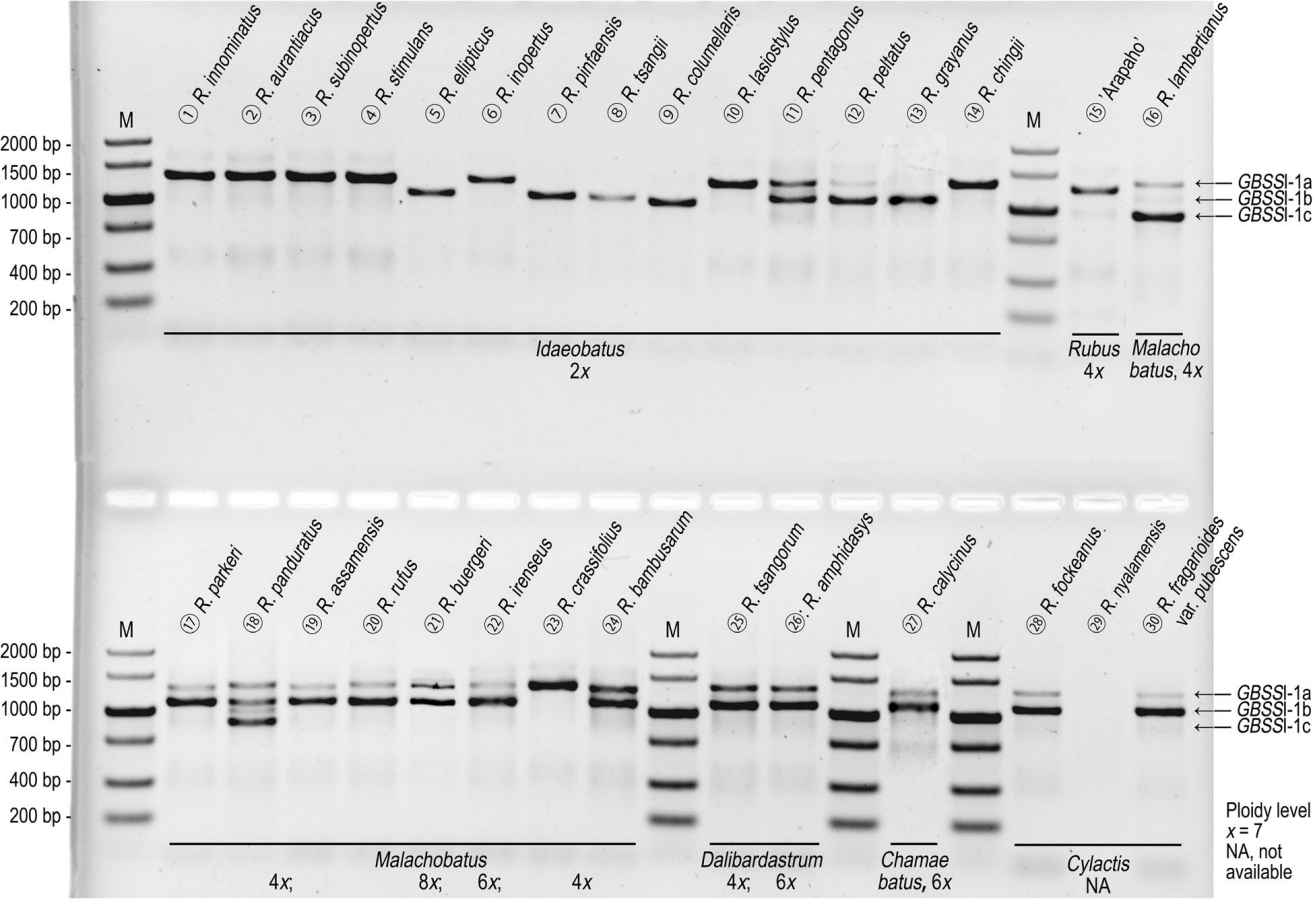
The number of *GBSS*I-1 variants within Chinese *Rubus* at different ploidy levels. The arrows represent the positions of individual *GBSS*I-1 variants

**Fig. 2:**
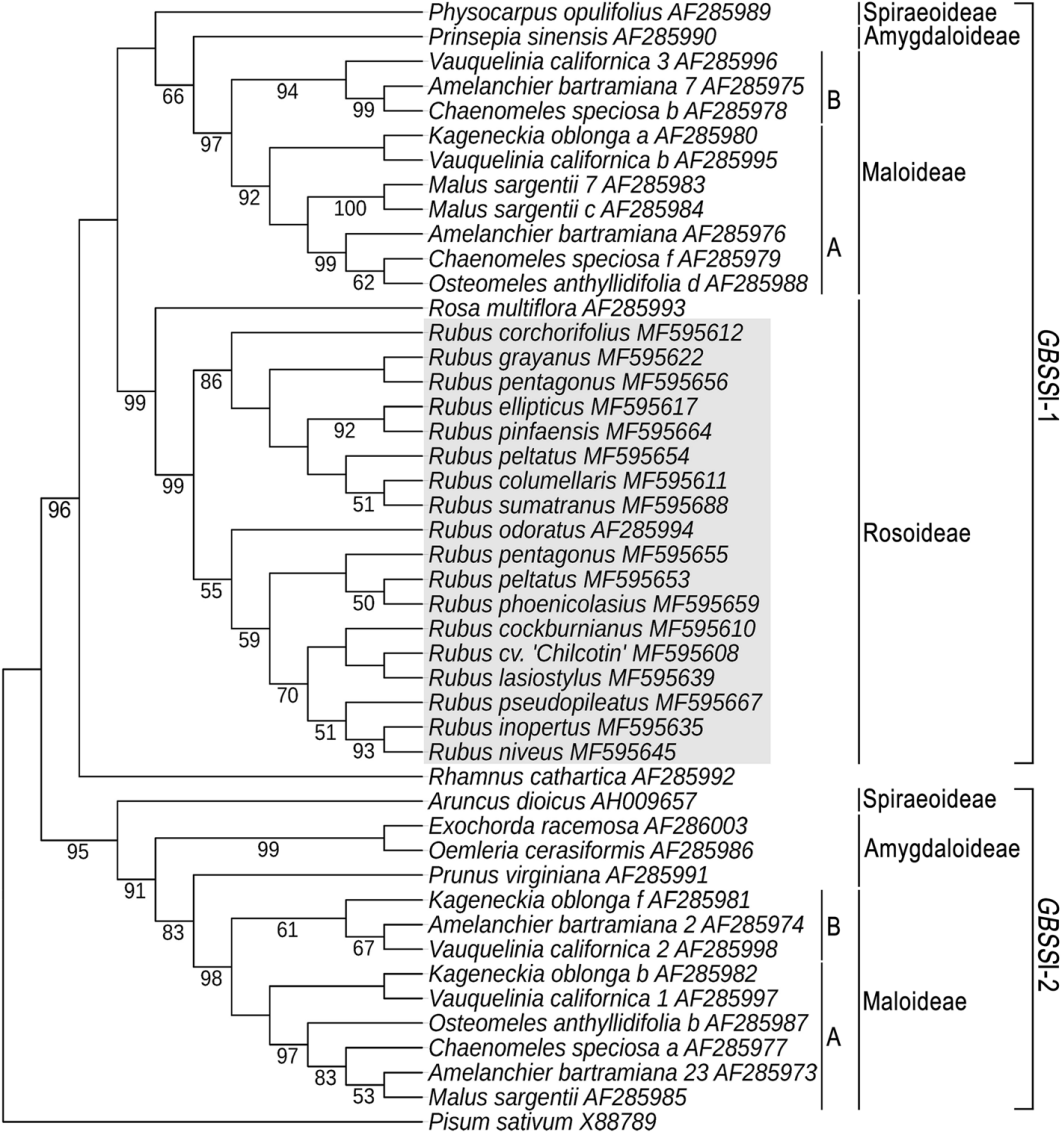
Neighbor-joining (NJ) tree obtained with *Rubus GBSS*I-1 exon sequence and corresponding published *GBSS*I-1 and *GBSS*I-2 sequences from Rosaceae subfamilies, rooted with *Pisum* and *Rhamnus* sequences. Brackets on right delimit groups of paralogous sequences, *GBSS*I-1 and *GBSS*I-2. Short vertical lines (A and B) indicate a second duplication of the *GBSS*I gene occurring in some species of subfamily Maloideae. *Rubus* sequences obtained in this study are underlined in grey. Bootstrap values obtained from 1000 replicates are shown under the branches

As shown in Fig. 1 and Additional file 2, we obtained different *GBSS*I-1 variants (*GBSS*I-1a, *GBSS*I-1b and *GBSS*I-1c) within *Rubus* at different ploidy levels. Based on the definition of ortholog by Yu et al. [32], we carried out the orthology assessment. The different *GBSS*I-1 variants shared > 90% identity at the amino acid sequence level with a significant E-value (< 10^− 10^), and distributed on the same zone of chromosome 7 by alignment with reference genome of diploid *R. occidentalis* L. [33] (Additional file 3). Orthology of the *Rubus* diploid sequences was also assessed using phylogenetic analysis. The dataset was obtained from our *GBSS*I-1 sequences from *Rubus* diploids and from *GBSS*I (1 and 2) coding region sequences of Rosaceae species available in GenBank. This matrix included 378 nucleotides sites, of which 141 were constant and 164 were phylogenetically informative. The phylogenetic tree (Fig. 2) grouped all the Rosaceae *GBSS*I sequences into two well-supported clades with bootstrap values of 96 and 95%, respectively. These clades represented paralogous genes, corresponding to *GBSS*I-1 and *GBSS*I-2 according to Evans et al. [29]. In the *GBSS*I-1 clade, all the *Rubus* diploid sequences fell in a well-supported clade (99% BS), which provided evidence that these sequences were orthologous.

For diploids, *GBSS*I-1a was detected in species of subsections *Thyrsidaei*, *Idaeanthi*, *Pileati*, and *Wushanenses*, and *Corchorifolii*, and most *Stimulantes* and *Pungentes* species (Fig. 1, ①-④, ⑥, ⑩, ⑭), while *GBSS*I-1b was detected in subsects. *Rosaefolii*, *Leucanthi*, and *Corchorifolii* (Fig. 1, ⑧, ⑨, ⑬), as well as *R. ellipticus* of subsect. *Stimulantes*, and *R. pinfaensis*, *R. macilentus* and *R. simplex* of subsect. *Pungentes* of sect. *Idaeobatus* (Fig. 1, ⑤, ⑦). Both *GBSS*I-1a and *GBSS*I-1b alleles were found in subsects. *Alepestres* and *Peltati* species (Fig. 1, ⑪, ⑫). Genotyping patterns varied among polyploids. Only one copy was observed in blackberry cultivar ‘Arapaho’ (4*x*) of sect. *Rubus* (Fig. 1, ⑮). Both *GBSS*I-1a and *GBSS*I-1b homoeologs were detected in polyploids including tetraploids, hexaploid, and octoploid of sect. *Malachobatus* (Fig. 1, ⑯, ⑰, ⑲-, ). *R. panduratus* had three alleles, *GBSS*I-1a, *GBSS*I-1b and *GBSS*I-1c (Fig. 1, ⑱), and *R. crassifolius* possessed only *GBSS*I-1a (sequence not obtained) (Fig. 1, ). There were two homoeologs (*GBSS*I-1a and -1b) in sects. *Dalibardastrum*, *Chamaebatus*, and *Cylactis* species (Fig. 1, -, band of *R. nyalamensis* not shown).

### Gene structure and sequence characteristics

**Fig. 3:**
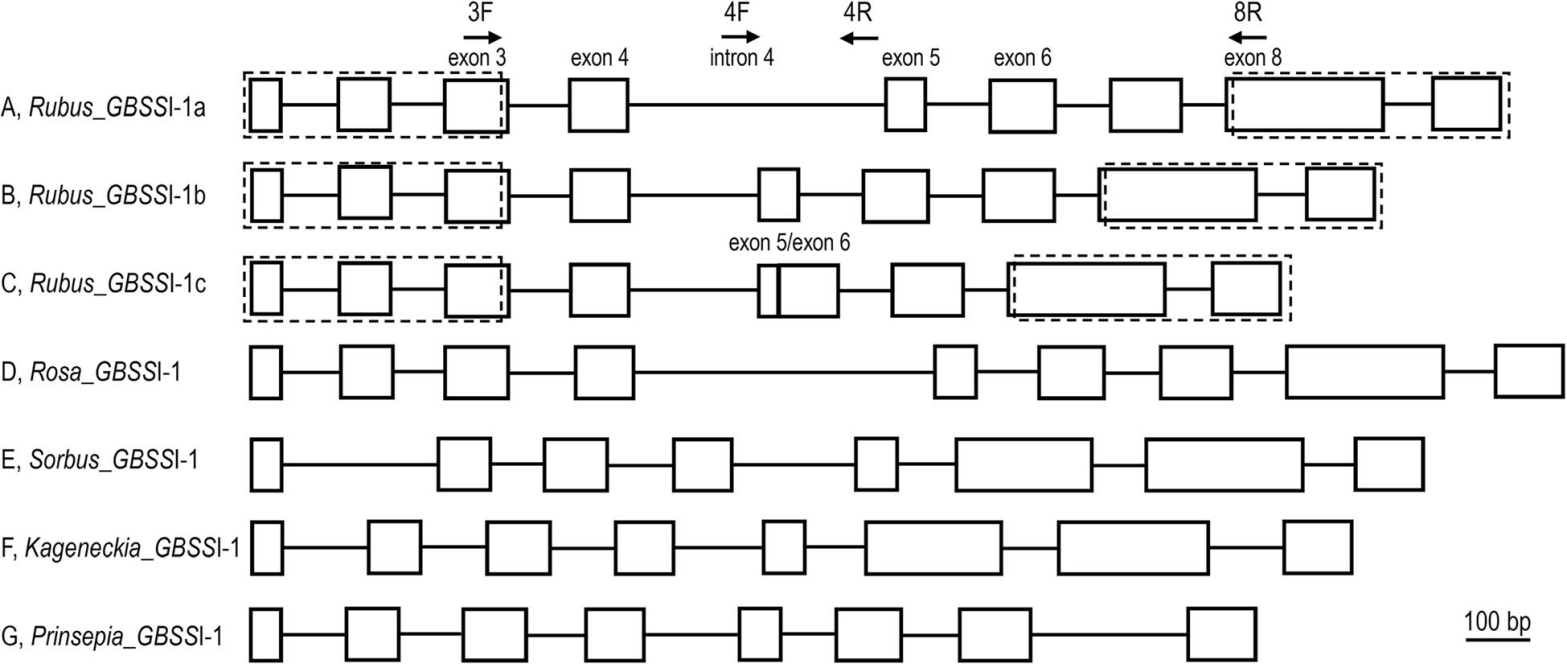
Schematic diagram of *GBSS*I-1 gene within the family Rosaceae. Open boxes represent exons, and connecting lines represent introns. The fourth intron of *GBSS*I-1b and *GBSS*I-1c was at least 260 bp shorter than *GBSS*I-1a, and an additional missing fifth intron was detected in *GBSS*I-1c. Arrows represent the locations and directions of primers used for PCR amplification. Rows A, B and C are different *GBSS*I-1 genes from *Rubus* obtained in this study; Rows D-G are *GBSS*I-1 sequences from *Rosa* (Rosoideae, AF285993), *Sorbus* (Maloideae, AF500468), *Kageneckia* (Spiraeoideae, DQ874892), and *Prinsepia* (Amygdaloideae, AF285990). The dashed box represents the fragment not obtained in this study

According to the gene structure and sequence divergence, three homoeologs representing *GBSS*I-1a, *GBSS*I-1b, and *GBSS*I-1c were identified (Fig. 3). *GBSS*I-1a (e.g., from *R. odoratus*, GenBank no. AF285994), had a classical *GBSS*I gene structure with eight introns (part of the full-length sequence). Similar structure was observed in *GBSS*I-1b and *GBSS*I-1c, but intron length varied between and within *GBSS*I-1a, −1b and -1c. The intron 4 of *GBSS*I-1b and *GBSS*I-1c was at least 260 bp shorter than *GBSS*I-1a. An additional missing intron 5 was detected in *GBSS*I-1c (Fig. 3, a-c). In addition, a longer 4th intron in Rosoideae *GBSS*I-1 (Fig. 3, a-d) was observed than other three subfamilies (Fig. 3, e-g), consistent with the results of Evans et al. [29].

After treating the gaps as missing data, we obtained 195 sequences for *GBSS*I-1 gene (Table 1). *GBSS*I-1a existd in 83 individuals whereas *GBSS*I-1b was found in 58 individuals. Three taxa containing five individuals possess *GBSS*I-1c. The final aligned *GBSS*I-1a consisted of 1296 nucleotides with length ranging from 1139 to 1234 base pairs. There were 441 (34.03%) variable characters, of which 257 (19.83%) were parsimony-informative. The aligned intron 4 was composed of 517 bp with length ranging from 403 to 484 bp, which had 188 variable sites. Seven indels were present in the entire gene alignment. The indels consisted of 1–303 nucleotides. Two relatively large ones (an insertion of 136 bp, and an insertion of 303 bp) were found in *GBSS*I-1a group.

**Table 1:** Sequence variations of *GBSS*I-1 homoeologs in *Rubus* (excluding outgroups)

Region	Number of individuals	Number of sequences	Length range (bp)	Aligned nucleotide length (bp)	Variable sites (%)	Parsimony informative sites (%)
*GBSS*I-1	140	195	760–1234	1325	583 (44.00%)	366 (27.62%)
*GBSS*I-1 intron	140	195	482–858	938	436 (46.48%)	273 (29.10%)
*GBSS*I-1a	83	118	1139–1234	1296	441 (34.03%)	257 (19.83%)
*GBSS*I-1a intron	83	118	763–858	917	340 (37.08%)	192 (20.94%)
*GBSS*I-1a intron 4	83	118	403–484	517	188 (36.36%)	106 (20.50%)
*GBSS*I-1b	58	72	942–1001	1028	234 (22.76%)	134 (13.04%)
*GBSS*I-1b intron	58	72	563–621	641	170 (26.52%)	98 (15.29%)
*GBSS*I-1b intron 4	58	72	191–249	252	65 (25.79%)	34 (13.49%)
*GBSS*I-1c	5	5	760–822	913	11 (1.20%)	–

The length of *GBSS*I-1b varied from 942 to 1001 bases. There were 234 (22.76%) variable sites, of which 134 (13.04%) were parsimony-informative in 1028 aligned nucleotides. The intron 4 contained 252 aligned nucleotides from 191 to 249 bp, and 65 variable sites. The alignment of the entire gene had four indels, each including 1 to 9 nucleotides. The aligned *GBSS*I-1c contained 913 bp with length range from 760 to 822 bp, of which just 11 were variable. JModelTest suggested that the best-fit model selected by Akaike Information Criterion (AIC) was TIM2 + G for *GBSS*I-1 dataset.

### Phylogenetic analysis

**Fig. 4:**
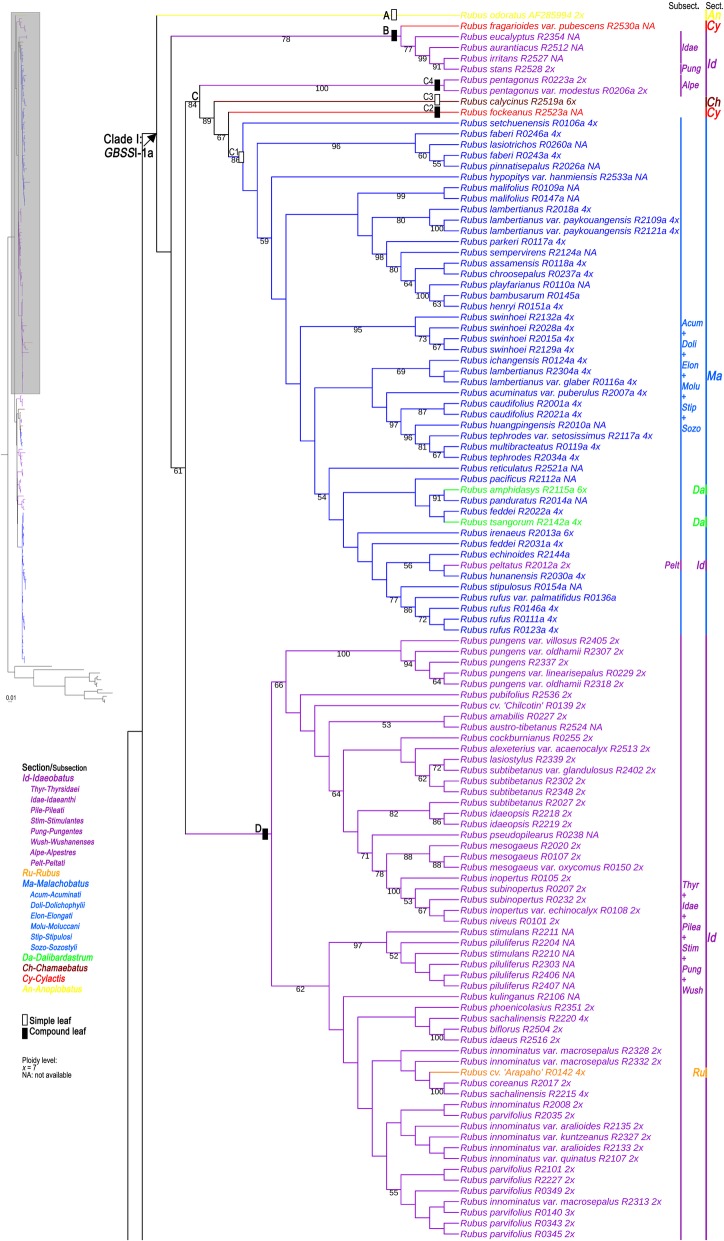
Maximum likelihood (ML) tree inferred from the *GBSS*I-1a sequences of *Rubus*. Bootstrap values >50 based on 1000 replicates are provided below the branches

**Fig. 5:**
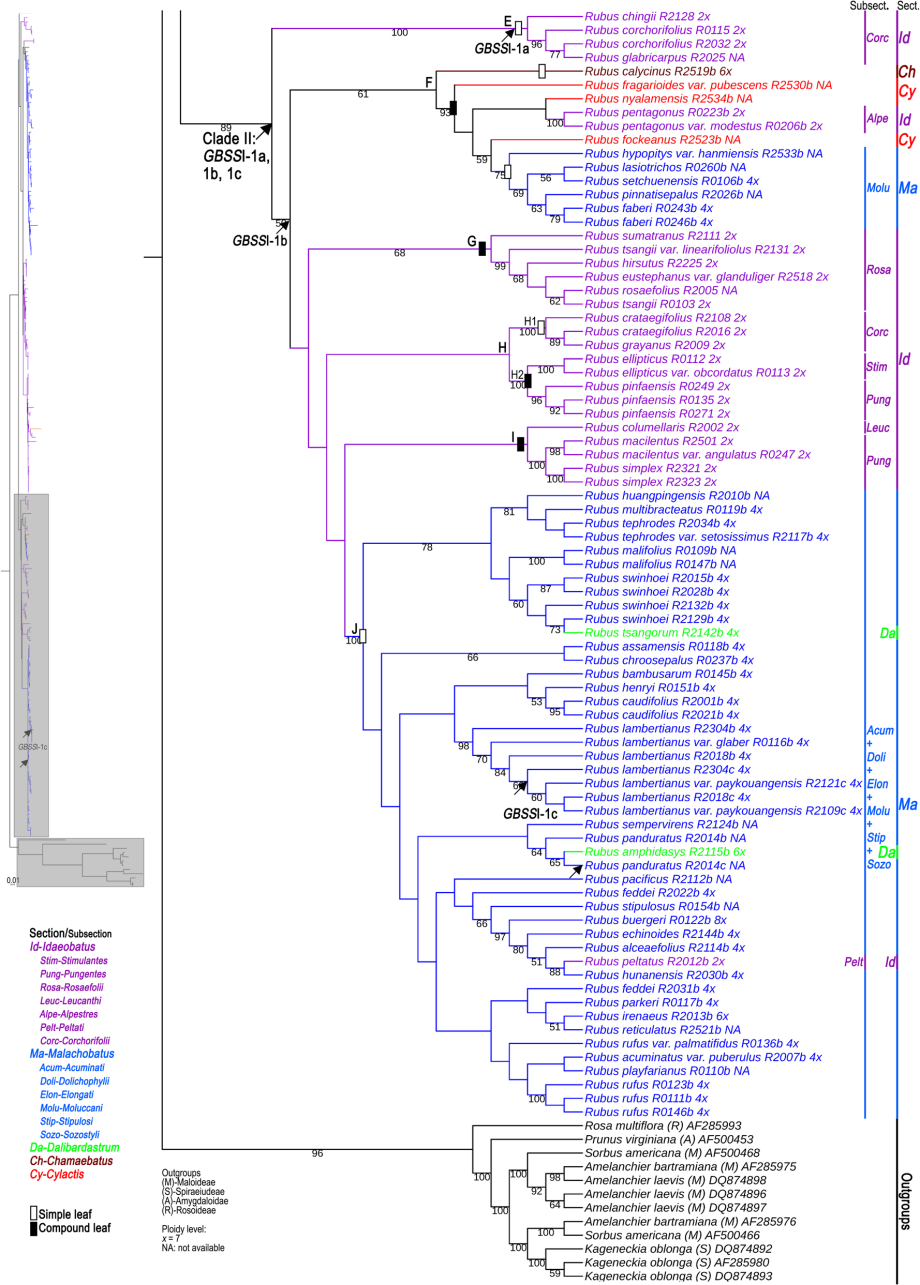
Maximum likelihood (ML) tree inferred from the *GBSS*I-1b/1c sequences of *Rubus*. Bootstrap values >50 based on 1000 replicates are provided below the branches

The *GBSS*I-1 gene tree generated by both Maximum Likelihood (ML) and Bayesian Inference (BI) analyses resulted in largely congruent tree topologies, suggesting two major lineages within *Rubus* (Figs. 4, 5, Additional files 4, 5). Clade I consisted of four subclades (A-D), corresponding to most taxa with *GBSS*I-1a. As shown in Fig. 4, subclades A and B were represented by *R. odoratus* of sect. *Anoplobatus*, *R. fragarioides* var. *pubescens* of sect. *Cylactis* and four sect. *Idaeobatus* species. All samples of sect. *Malachobatus*, and sect. *Dalibardastrum*, as well as *R. peltatus* of subsect. *Peltati* from sect. *Idaeobatus* formed a monophyletic group (C1) with high support values (86% BS, 1.00 PP). *Rubus fockeanus* (C2) from sect. *Cylactis*, *R. calycinus* (C3) from sect. *Chamaebatus*, and *R. pentagonus* (C4) from subsect. *Alpestres* of sect. *Idaeobatus* (C4) and C1 were sister to each other. The four groups formed a well-supported (84% BS, 1.00 PP) subclade C. Subclade D included species of subsections *Thyrsidaei*, *Idaeanthi*, *Pileati*, and *Wushanenses*, and most *Stimulantes* and *Pungentes* from sect. *Idaeobatus* without clear circumscription among subsections based on traditional taxonomy (0.76 PP). Blackberry cultivar ‘Arapaho’ of sect. *Rubus* was nested within the subclade D.

Clade II was divided into six subclades (E-J), corresponding to all taxa with *GBSS*I-1b/1c as well as four taxa with *GBSS*I-1a (Fig. 5). The remaining sect. *Idaeobatus* species were mainly clustered into four subclades (E, G, H, and I). The subsect. *Corchorifolii* taxa dispersed in the two groups E and H1 with *GBSS*I-1a and -1b, respectively. Group H2 consisted of *R. ellipticus* from subsect. *Stimulantes* and *R. pinfaensis* of subsect. *Pungentes*. Subclade G corresponded to subsect. *Rosaefolii* species (68% BS, 1.00 PP). Subsect. *Leucanthi* species and *R. macilentus*, *R. simplex* of subsect. *Pungentes* formed subclade I (0.69 PP). Subclade F included taxa from sects. *Chamaebatus*, *Cylactis*, and *R. pentagonus* of subsect. *Alpestres* from sect. *Idaeobatus*, as well as six taxa of subsect. *Moluccani* from sect. *Malachobatus*. Well-supported (100% BS, 1.00 PP) subclade J was composed of most sect. *Malachobatus* taxa with *GBSS*I-1b and three taxa with *GBSS*I-1c, which was almost consistent with group B1 (Fig. 4).

### Phylogenetic network

**Fig. 6:**
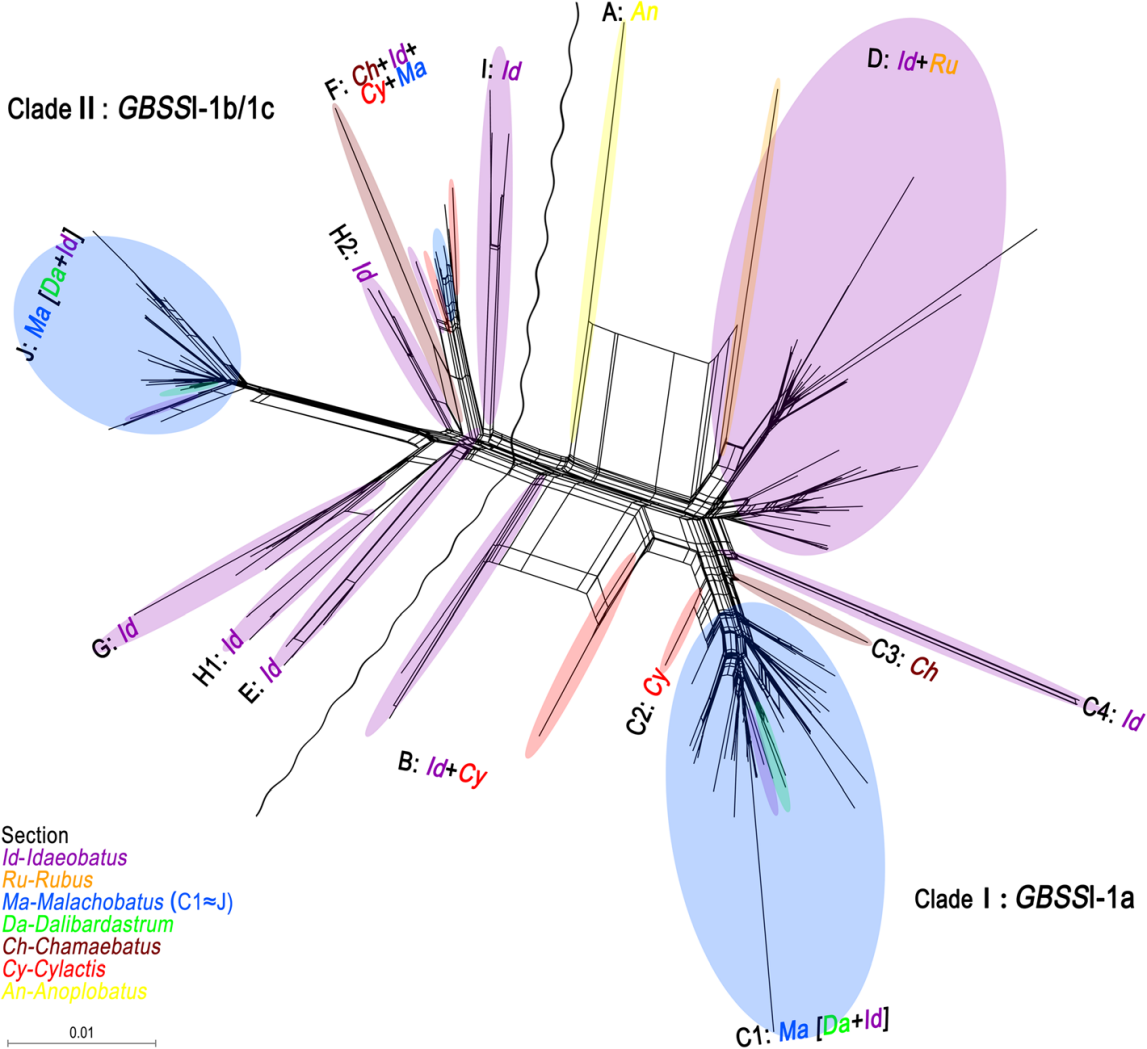
Neighbor-Net diagram based on uncorrected-*P* distances of *GBSS*I-1 DNA sequences of *Rubus*. The wavy lines represent the discrimination of two main sequences of *GBSS*I-1 gene. Major branches indicated >90 bootstrap values (data not shown). The capital letters correspond to the lineages of Figs. 4 and 5

A neighborNet diagram (Fig. 6) showed the same general patterns as the phylogenetic tree, corresponding to *GBSS*I-1a and *GBSS*I-1b/1c of the *GBSS*I-1 sequences in the two splits. The *GBSS*I-1a sequences could distinguish four broad groups: group A (corresponding to the major sect. *Idaeobatus* subclade in Fig. 4), group B (corresponding to sects. *Malachobatus* (*Dalibardastrum* + subsect. *Peltati*) - *Cylactis* - *Chamaebatus* - subsect. *Alpestres* subclade), group C (minor *Idaeobatus*-*Cylactis* subclade), and *Anoplobatus* group D. *GBSS*I-1b was occupied by species of the lineages E-J in Fig. 5.

## Discussion

### Orthologs of *GBSS*I-1 gene in *Rubus*

Orthology assessment is an important concern when using nuclear genes to reconstruct phylogeny, since paralogous sequences may lead to erroneous phylogenetic inferences [34, 35]. We carried out sequence alignment and phylogenetic analysis to test the orthology and paralogy of *GBSS*I-1. Rousseau-Gueutin et al. [26] hypothesized orthology of the *DHAR* sequences because they shared similar positions in both diploid and the cultivated octoploid strawberry genomes. The *GBSS*I-1 sequences from *Rubus* shared the same location among different genomes (Additional file 3). From the phylogenetic analysis (Fig. 2), we observed the *Rubus* sequences belonged to the same gene copy, *GBSS*I-1, which supported their orthologous status.

Compared with single copy *GBSS*I-2 in *Rubus* [25], *GBSS*I-1 gene was complex within the genus. Either *GBSS*I-1a or *GBSS*I-1b was detected in most diploids, while both of them were detected in *R. pentagonus* and *R. peltatus*, indicating their probable interspecific hybrid origin. Interestingly, different orthologs were identified based on gene structure within subsect. *Corchorifolii* of sect. *Idaeobatus* (Fig. 1, Additional file 2). Four taxa had *GBSS*I-1a and the other three had *GBSS*I-1b, which were clustered into subclades E and H1, respectively (Fig. 5). The two subclades belonged to clade II in gene trees, incongruent with their structure difference. We speculated that the *GBSS*I-1b originated from *GBSS*I-1a in some diploids by mutation. The two homoeologs also existed in majority of polyploids of sects. *Malachobatus*, *Dalibardastrum*, *Chamaebatus*, and *Cylactis* (unknown ploidy levels). Several sect. *Malachobatus* species even had *GBSS*I-1a, *GBSS*I-1b and *GBSS*I-1c. Tetraploid *R. crassifolius* (sect. *Malachobatus*) and blackberry cultivar ‘Arapaho’ (sect. *Rubus*) were exceptions with just one copy.

Of the 195 *GBSS*I-1 sequences in this study, seven contained stop codons and might have become pseudogenes, containing *GBSS*I-1a in *R. fragarioides* var. *pubescens*, *GBSS*I-1b in *R. lambertianus* and five *GBSS*I-1c sequences in *R. lambertianus*, *R. lambertianus* var. *paykouangensis* and *R. panduratus* (Additional file 2). All of them had deletions or insertions in the exon regions, leading to the nonsense mutation. The five *GBSS*I-1c sequences, with the missing fifth intron, might have become pseudogenes, but they might raise in quite recent since they had not yet led to long branches (the brief phylogram in the upper left corner in Fig. 5). Phylogenetic tree revealed that *GBSS*I-1c sequences were nested within *GBSS*I-1b clade (Fig. 5). It was reasonable to conclude that the *GBSS*I-1c type was directly originated from *GBSS*I-1b by mutation. Intron losses had been found in *GBSS*I-1 genes of diverse taxa, like subfamily Maloideae [29, 31] and Pooideae [36]. In some species of Poeae, the *GBSS*I intron loss was interpreted as a nonhomoplasious synapomorphy [36]. Hu [37] proposed the ‘intron exclusion hypothesis’, which suggested that a single intron could be precisely removed by double strand breaks (DSB) from a multiple-intron gene. This model of intron loss may explain the present results.

### Incongruence between *GBSS*I-1-based phylogeny and traditional *Rubus* classification

Overall, *GBSS*I-1-based phylogeny largely supported Yü’s rather than Focke’s taxonomy. The results also generated some conflicts with the traditional morphology-based taxonomy, consistent with our previous study by chloroplast and single copy nuclear genes [25]. These incongruences probably suggested the need for a taxonomic revision using modern approaches.

The taxonomic treatments of *R. ellipticus*, *R. ellipticus* var. *obcordatus*, and *R. pinfaensis* have long been fraught with controversy. The dispute has mainly focused on two aspects, whether *R. ellipticus* and *R. pinfaensis* should be combined or not, and *R. ellipticus* var. *obcordatus* should be treated as a species *R. obcordatus* or a variety of *R. ellipticus* [2, 5, 6, 38–40]. In terms of character differences, *R. ellipticus* has dense pubescentia in blade back and *R. pinfaensis* has sparse villus [5]. On the contrary, the differences between *R. ellipticus* and *R. ellipticus* var. *obcordatus* not only focus on the leaflet shape and size, but also on the growth habits and habitat, inflorescence and flowering time [39]. Moreover, significant differences also exhibited in the pollen features, rDNA chromosomal distribution and genomic relationships by molecular cytogenetics [12, 39, 40]. In this study, three *R. pinfaensis* samples formed a strongly supported clade with the cluster of *R. ellipticus* and *R. ellipticus* var. *obcordatus*. The clade revealed obvious genetic divergence with any other species from both subsects. *Stimulantes* and *Pungentes* (Fig. 5, Additional file 5). Therefore, we supported to place them into a separate series *Elliptici*, sect. *Idaeanthi*, subg. *Idaeobatus*, as Focke proposed [2].

*Rubus simplex* was firstly placed into series *Saxatiles* of subg. *Cylactis* by Focke [1], while Yü et al. [5] and Lu & Boufford [6] moved it into subsect. *Pungentes* of sect. *Idaeobatus* because its stipules adnate to base of petioles. Our phylogenies revealed that *R. simplex* formed a cluster with *R. macilentus* of sect. *Idaeobatus* rather than with sect. *Cylactis* species (Fig. 5, Additional file 5), partly supporting the traditional taxonomic treatment by Yü et al. and Lu [5, 6]. However, this cluster formed a clade with *R. columellaris* of subsect. *Leucanthi*, which exhibited deep divergence with other species of subsect. *Pungentes* (Fig. 4, Additional file 5). Thus, subsect. *Pungentes* was clearly demonstrated to be polyphyletic.

*Rubus peltatus* (2*n* = 2*x* = 14) possesses some unique characters, such as peltate simple leaves, ovate stipules and 1-flowered with 5 cm or more in diameter, but distinct from other species of sect. *Idaeobatus* [5, 6, 41]. Both Species *Ruborum* [1] and Flora of China [5, 6] separately assigned it into subsect. *Peltati* of sect. *Idaeobatus*. *Rubus peltatus* revealed *GBSS*I-1a and -1b alleles, congruent with most tetraploid *Malachobatus* species. Here, it formed a moderately supported clade with some subsect. *Moluccani* species of sect. *Malachobatus* (Figs. 4, 5, 6). This suggested that *R. peltatus* might be closely related to polyploids. Moreover, diploid species of *R. fulvus*, *R. micropetalus*, and *R. paniculatus* have been reported to occur in the predominantly polyploid sect. *Malachobatus* [42–44]. Its rational taxonomic position needs to be explored further by multiple researches.

### Allopolyploid origin of *Rubus* polyploids

Hybridization is believed to play an important role in plant speciation and evolution [24]. Chromosome numbers provide preliminary evidence for the possible hybrid origin of the sect. *Malachobatus*. The majority of the species from the sect. *Idaeobatus* present the chromosome number of 2*n* = 2*x* = 14 [9]. On the other hand, species in the sects. *Malachobatus*, *Dalibardastrum* and *Chamaebatus* have been reported to have higher ploidy levels (e.g., 2*n* = 4*x* = 28 for most species; *R. amphidasys*, 2*n* = 6*x* = 42; *R. buergeri*, 2*n* = 8*x* = 56) [9]. It is predicted that many speciation events in *Rubus* are associated with a change in ploidy levels. Thus, polyploidization may have played an important evolutionary role in the origin of the three sections. This study further offered the potential for new insights into the allopolyploid origin, especially in sect. *Malachobatus*.

#### Section *Malachobatus*

In our previous studies, bivalent pairing was the most predominant form in meiotic configuration, with just very few multivalents in some *Malachobatus* polyploids [13]. Moreover, polymorphism of 45S rDNA signal intensities by FISH were detected among them, implying different repeat copy numbers among different rDNA sites [12]. These results suggested that some sect. *Malachobatus* species be probable of allopolyploid origin. Here, *GBSS*I-1 homoeologs from the same polyploid individual dispersed in different well-supported clades in the *GBSS*I-1 gene tree (Figs. 4, 5, 6, Additional file 5), and some of these homoeologs were more closely related to homoeologs in other species than they were to each other, indicating that the homoeologs were donated by different ancestral taxa. As Wendel & Doyle [45] and Fortune [46] proposed, the sequences duplicated by polyploidy should be each other’s closest clades in autopolyploids, whereas be distributed in different clades in allopolyploids. This mechanism has been clearly illustrated in the origin of allotetraploid rice by Ge et al. [47]. Therefore, our findings provided strong evidence for allopolyploid origin of most sect. *Malachobatus* species. This hypothesis indicated that two kinds of diploids hybridized to form most allotetraploid species.

#### Section *Dalibardastrum*

Section *Dalibardastrum* species are also allopolyploids because of the co-occurrence of *GBSS*I-1a and -1b homoeologs. *Rubus tsangorum* and *R. amphidasys* share some morphological similarities, such as weak, densely bristly, prostrate stems, simple leaves, and terminal or axillary inflorescences, subracemes with 5 to 15 flowers, whereas they were reported as a tetraploid and hexaploid, respectively [9]. Both of them were strongly nested within sect. *Malachobatus* group (Figs. 4, 5, 6, Additional file 5), which suggested that they share parental ancestors from sect. *Malachobatus*. In addition, no other homoeologs besides *GBSS*I-1a and -1b were found in the hexaploid. As a consequence, the hexaploid might be derived from tetraploid without further hybridization, but only through unreduced gamete of tetraploid (4*x* and 2*x*).

#### Section *Cylactis*

Members of sect. *Cylactis* formed a clearly polyphyletic group (Figs. 4, 5, 6, Additional file 5). They are creeping herbs with 3- or 5-foliolate compound leaves and several flowers in clusters or solitary [6]. This section contains various ploidy levels with diploid, tetraploid, and mixoploid [9]. Unfortunately, chromosome numbers of the examined taxa have never been reported. They all have two alleles of the *GBSS*I-1 gene, suggesting that hybridization events may have been involved in the origin. Specifically in sect. *Cylactis*, apomixis has also been found [48], hence various ploidy levels may be generated.

### The role of diploid sect. *Idaeobatus* in the evolution within *Rubus*

Diploid sect. *Idaeobatus* is one of the largest sections in *Rubus*, which has been resolved as a polyphyletic group with several different evolutionary routes [25]. Here, *GBSS*I-1-based phylogeny strongly support our previous results (Figs. 4, 5, 6, Additional file 5). This was congruent with its morphological diversity [5, 6]. The majority of diploids with *GBSS*I-1a are composed of imparipinnately 3–9(− 11)-foliolate leaves and flowers in mainly corymbs, while subsect. *Corchorifolii* with *GBSS*I-1a consist of simple leaves in 1-flowered, and the remaining diploids with *GBSS*I-1b with imparipinnately 3–5(− 9)-foliolate or simple leaves and flowers in subracemes. Particularly, *R. pentagonus* and *R. peltatus* with both *GBSS*I-1a and -1b is solitary flower with relative large diameter, with palmately 3-foliate and simple leaves, respectively. Furthermore, *Idaeobatus* species exhibit both sexual and asexual reproduction, and some species could freely hybridize with each other and produce fertile offspring [15–17, 19]. This probably contribute to the formation of new species, among which polyploids are contained.

Based on the structure difference and phylogeny, *GBSS*I-1b originated from *GBSS*I-1a in some diploids by mutation, then polyploidization happened between species with *GBSS*I-1a and -1b. Therefore, to some extent, the early-divergent diploid species with *GBSS*I-1a or -1b emerged before polyploid formation in the evolution of *Rubus*. Then they probably experience their own distinct evolutionary history with various evolutionary rates [25]. During the process, various but common diploidization events might occur in these polyploids [24], hence the allotetraploid is the most frequent and stable form within *Rubus* [9].

## Conclusions

This study presented phylogenies of genus *Rubus* based on low-copy nuclear *GBSS*I-1 gene with a comprehensive taxon sampling with 140 *Rubus* individuals representing 102 taxa in 17 (out of the total 24) subsections of 7 (total of 12) sections at different ploidy levels. Either *GBSS*I-1a or *GBSS*I-1b was detected in most diploids (except for *R. pentagonus* and *R. peltatus* with both two alleles) of sect. *Idaeobatus* and blackberry cultivar of sect. *Rubus*. Both homoeologs (1a and 1b) were observed in majority of polyploids from sect. *Malachobatus*, as well as in sects. *Dalibardastrum*, *Chamaebatus*, and *Cylactis* species. Phylogenetic trees showed two clades I and II, corresponding to *GBSS*I-1a, and *GBSS*I-1b/1c sequences. *GBSS*I-1 homoeologs from the same polyploid individual dispersed in different well-supported clades in the *GBSS*I-1 gene tree, and some of these homoeologs were more closely related to homoeologs in other species than they were to each other, indicating that the homoeologs were donated by different ancestral taxa. Based on the structure difference and phylogeny, *GBSS*I-1b originated from *GBSS*I-1a in some diploids by mutation, then polyploidization happened between species with *GBSS*I-1a and -1b. Two kinds of early-divergent ancestral diploids hybridized to form most extent allotetraploid species. This study provided new insights into allopolyploid origin and evolution from diploid to polyploid within genus *Rubus* at the molecular phylogenetic level, consistent with the taxonomic treatment by Yü et al. and Lu.

## Methods

### Taxa sampling

The *Rubus* classification of this study follows the system used in recent floristic treatments by Yü et al. [5] and Lu & Boufford [6], since the majority of species sampled here are native in China. In total, we sampled 139 *Rubus* individuals, of which 85 (representing 59 taxa) are from 11 subsections of sect. *Idaeobatus*, one from sect. *Rubus*, 47 (representing 36 taxa) from 6 out of 13 subsections of sect. Malachobatus, two from sect. *Dalibardastrum*, one from sect. *Chamaebatus*, and three from sect. *Cylactis* (Additional file 2). These samples, with confirmed ploidy level, include 68 diploids (2*n* = 14), one triploid (2*n* = 21), 37 tetraploids (2*n* = 28), three hexaploids (2*n* = 42), and one octoploid (2*n* = 56) (Additional file 2) [9–11, 49–51]. Voucher specimens were deposited in the herbarium for horticultural plants, Sichuan Agricultural University (This herbarium is not indexed). *Rubus odoratus* (2*n* = 14) [49] of subgenus *Anoplobatus* (almost corresponding to section by Yü) was also included in this study. Some representative species from family Rosaceae were selected as outgroups (Additional file 2).

### DNA isolation, amplification, cloning and sequencing

Genomic DNA was extracted from silica-gel dried or frozen leaf tissues following the modified cetyltrimethyl ammonium bromide (CTAB) method [52]. Primers 3F (5′-TAC AAA CGA GGG GTT GAT CG-3′) and 8R (5′-GAT TCC AGC TTT CAT CCA GT-3′) [30] were used to amplify *GBSS*I-1 gene. Primers 4F (5′-ACA AGA GGC AGC ATT AWA CAT CAG-3′) and 4R (5′-GGA AMC AAA AAG AGA GAA TCG GTA AGG-3′) were designed here to sequencing the long 4th intron of *GBSS*I-1. The amplified fragment comprises 7 bp at the 3′ end of the third exon, four complete exons, five complete introns, and 7 bp from the 5′ end of the eighth exon.

PCR amplification was performed in a PTC-200 thermocycler (Bio-rad, Hercules, CA). A volume of 25 μL amplification mixture contains 20 ng of template DNA, 2.5 μL of 10 × PCR buffer (10 mmol·L^− 1^ pH 8.0 Tris-HCl, 50 mmol·L^− 1^ KCl, 1.5 mmol·L^− 1^ EDTA), 1.2 μL of MgCl_2_ (25 mmol·L^− 1^), 1.4 μL of dNTP mix (10 mmol·L^− 1^), 1 μL of each primer (5 μmol·L^− 1^), and 1.5 U of PfuDNA polymerase (Tiangen, Beijing). The cycling programme began with an initial pre-denaturation at 94 °C for 4 min, followed by 30 cycles at 94 °C for 45 s, 55 °C for 1 min and 72 °C for 1.5 min. PCR finished after a final extension at 72 °C for 20 min.

PCR products were verified in a 1% agarose gel, and the target products were separated and purified by UNIQ-10 Column MicroDNA Gel Extraction Kit (Sangon, Shanghai, China). For diploids, purified products were directly sequenced with BigDye 3.1 reagents on an ABI PRISM 3730 automatic sequencer (Applied Biosystems, Foster City, California, USA) from both directions. Special attention was paid to those sites with overlapping peaks in the chromatograms, because they may indicate intra-individual variation (polymorphisms) [53]. If an obviously overlapping signal was detected in both the forward and reverse chromatograms, the site was considered to be putatively polymorphic between alleles or copies. Those products with polymorphic sites were cloned using TA cloning after A-tailing and ligated to pMD20-T vector with a kit (Takara, Dalian, China). More than three clones per sample were sequenced using M13^+^, M13^−^ primers. For polyploids and *R. peltatus*, *R. pentagonus*, two or more amplification bands were cloned separately to obtain sequences. All the sequences have been submitted to the GenBank database with accession numbers of MF595603-MF595796 (Additional file 2). In addition, *GBSS*I-1 sequences of *R. odoratus* and other Rosaceae species were downloaded from GenBank (Additional file 2) [29, 31, 54].

### Orthology identification

To identify the orthology of *GBSS*I-1 gene sequences, we conducted gene sequence similarities and performed phylogenetic analysis. According to Yu’s [32] definition of ortholog, the identity at the amino acid sequence level was employed by alignment with the reference genome of diploid *R. occidentalis* L. [33]. Sequence orthology analysis was also confirmed by phylogenetic analysis using exon sequences of the two *GBSS*I copies published from Rosaceae [29] together with corresponding sequences generated in this study from diploid *Rubus*. Sequences from *Pisum sativum* [55] and *Rhamnus catharticus* [29] were used as outgroups.

### Phylogenetic analyses

We used CLC Genomics Workbench v7.5 (CLC bio, Qiagen, Boston, MA) for sequence editing and assemblying. The boundaries between exons and introns were determined by aligning with *GBSS*I-1 sequence of *R. odoratus* [29] and preservations of the ‘GT’ and ‘AG’ at two ends of introns. Sequences were aligned with Muscle [56] and manually adjusted in the Molecular Evolutionary Genetics Analysis software (MEGA 7.0) [57] with gaps treated as missing data. Sequence variation within and between different homoeologs was calculated by MEGA 7.0.

The obtained sequences from all species were first blasted (BlastN) against the released *Rubus occidentalis* to confirm that they are derived from the same *GBSS*I-1 locus. For those species with two or more forms of amplicons, all cloned and sequenced sequences were included in multisequence alignment in MEGA (v7.0) to genotype the patterns. Since all sequences despite of various length exclusively hit the *GBSS*I-1 region, they were treated as different alleles from the same gene of *GBSS*I-1. Three major variants denoted as *GBSS*I-1a, *GBSS*I-1b, and *GBSS*I-1c were obtained and all analyzed in phylogeny reconstruction. If two or more homoeologs were detected in one species, all of them were included for this species. The best fitting substitution model for *GBSS*I-1 was determined with the Akaike Information Criterion (AIC) [58] using JModelTest v2.1.1 [59]. The maximum likelihood (ML) tree was conducted using IQ-TREE v1.4.2 [60, 61]. One thousand regular bootstrap replicates were performed to obtain confidence values for the branches. Bayesian inference (BI) was performed with MrBayes v3.2.1 [62]. The Markov chains Monte Carlo (MCMC) algorithm was run for 6,000,000 generations with one cold and three heated chains, at sample frequency of 100. The first 1,500,000 generations were discarded as burn-in. Clade posterior probabilities (PP) were calculated from the combined sets of trees. All tree visualizations and annotations were achieved with iTOL v3 (Interactive Tree Of Life) online tool [63].

Phylogenetic networks can reflect the conflicting evolutionary signals and highlight reticulate evolution. Here, a network was constructed for the *GBSS*I-1 dataset with SplitsTree 4.14.2, using a NeighborNet diagram based on uncorrected-*P* distance matrix [64]. Bootstrap support was estimated with 1000 replicates.

## Additional files

Additional file 1: Survey on the species number and ploidy levels of *Rubus* taxonomy. (DOCX 93 kb)

Additional file 2: List of studied *Rubus* taxa, herbarium information, ploidy level, locality, and GenBank accession numbers of *GBSS*I-1 variants, and outgroups from family Rosaceae in this study. (DOCX 130 kb)

Additional file 3: The identity and E-value in *GBSS*I-1 of *Rubus* species by alignment with reference genome of diploid *R. occidentalis* L. (DOCX 96 kb)

Additional file 4: The simplified ML tree corresponding to Figs. 3 and 4 in *Rubus*. (JPG 2328 kb)

Additional file 5: Bayesian Inference (BI) tree inferred from the *GBSS*I-1 sequences of *Rubus*. Posterior probabilities >0.50 are shown below the branches. (JPG 7886 kb)

## Data Availability

The data sets supporting the conclusions of this article are included within its additional files.

## Abbreviations

BI: Bayesian inference
*GBSS*I: Granule-bound starch synthase I
LCNG: Low-copy nuclear gene
ML: Maximum likelihood
