# Supplementary material for: Allopolyploid origin in Rubus (Rosaceae) inferred from nuclear granule-bound starch synthase I (GBSSI) sequences
Source: BMC Plant Biol. 2019 Jul 10;19:303. doi: 10.1186/s12870-019-1915-7 (PMC6617891; doi:10.1186/s12870-019-1915-7)
Supplement: Supplementary file 1 — Survey on the species number and ploidy levels of Rubus taxonomy. (DOCX 93 kb) [file 12870_2019_1915_MOESM1_ESM.docx]

**Additional file 1** Survey on the species number and ploidy levels of *Rubus* taxonomy

| *Rubus* taxonomy by Focke^a-c^ | | *Rubus* taxonomy by Yü & Lu^d-e^ | | Ploidy level^f-k^ | Taxa used in this study |
| --- | --- | --- | --- | --- | --- |
| Subgenus / Section /  Subsection or Series | Number of  species | Section / Subsection | Number of  species |  |  |
| **Ⅰ Subg. *Chamaemorus*** | 1 | **8 Sect. *Chamaemorus*** | 1 | 6*x*, 8*x* |  |
| **Ⅱ Subg. *Dalibarda*** | 5 | — | — | NA |  |
| **Ⅲ Subg. *Chamaebatus*** | 5 | **6 Sect. *Chamaebatus*** | 5 | 2*x*, 6*x* | 1 |
| **Ⅳ Subg. *Comaropsis*** | 2 | — | — | 4*x* |  |
| **Ⅴ Subg. *Cylactis***  4 Series | 14 | **7 Sect. *Cylactis*** | 9 | 2*x*, 3*x*, 4*x* | 3 |
| **Ⅵ Subg. *Orobatus*** | 19 | — | — | 6*x* |  |
| **Ⅶ Subg. *Dalibardastrum*** | 4 | **5 Sect. *Dalibardastrum*** | 11 | 4*x*, 6*x* | 2 |
| **Ⅷ Subg. *Malachobatus***  7 Sections and 7 Series | **114** | **4 Sect. *Malachobatus***  13 Subsections | **92** | 4*x* (Predominate  class), 6*x*, 8*x*, 14*x* | 36 taxa (47 individuals)  in 6 of 13 subsections |
| **Ⅸ Subg. *Anoplobatus*** | 6 | — | — | 2*x* | 1 (from GenBank) |
| **Ⅹ Subg. *Idaeobatus***  10 Sections and 7 Series | **117** | **1 Sect. *Idaeobatus***  11 Subsections | **88** | 2*x* (Predominate  class), 3*x*, 4*x* | 59 taxa (85 individuals)  in 11 subsections |
| **Ⅺ Subg. *Lampobatus*** | 10 | **2 Sect. *Lampobatus*** | 1 | NA |  |
| **Ⅻ Subg. *Rubus***  6 Sections and 6 Subsections | **444**^l^ | **3 Sect. *Rubus*** | 1 | 2*x*-9*x* | 1 (Blackberry cultivar) |
| Total | **≥741** |  | **≥208** |  |  |

Note: ^a-c^Focke (1900, 1911, 1914) [1-3], ^d^Yü et al., 1985 [5], ^e^Lu & Boufford, 2003 [6]; ^f^Thompson (1997) [9]; ^g^Wang et al. (2008) [11]; ^h^Naruhashi et al. (2002) [10]; ^i^Amsellem et al. (2001) [50]; ^j^Meng & Finn (2002) [51]; ^k^Thompson (1995) [49]; NA, not available.

^l^ There are greatly controversial on species numbers in subg. *Rubus* (sect. *Rubus*) due to frequent natural and artificial hybridization preference that caused species boundaries blurred.

The arrangement of sections of Yü's taxonomy is presented in a reverse order to those of Focke's system.
