# Supplementary material for: Allopolyploid origin in Rubus (Rosaceae) inferred from nuclear granule-bound starch synthase I (GBSSI) sequences
Source: BMC Plant Biol. 2019 Jul 10;19:303. doi: 10.1186/s12870-019-1915-7 (PMC6617891; doi:10.1186/s12870-019-1915-7)
Supplement: Supplementary file 2 — List of studied Rubus taxa, herbarium information, ploidy level, locality, and GenBank accession numbers of GBSSI-1 variants, and outgroups from family Rosaceae in this study. (DOCX 130 kb) [file 12870_2019_1915_MOESM2_ESM.docx]

**Additional file 2** List of studied *Rubus* taxa, herbarium information, ploidy level, locality, and GenBank accession numbers of *GBSS*I-1 variants, and outgroups from family Rosaceae in this study

| **Taxa** | **No. of**  **individuals** | **Herbarium voucher** | **Ploidy**  **level** | **Locality** | ***GBSS*I-1 variants** | | |
| --- | --- | --- | --- | --- | --- | --- | --- |
|  |  |  |  |  | ***GBSS*I-1a** | ***GBSS*I-1b** | ***GBSS*I-1c** |
| **Section *Idaeobatus*** | **85** |  |  |  |  |  |  |
| **1. Subsection *Thyrsidaei* (Focke) Yü et Lu** | **11** |  |  |  |  |  |  |
| *R. cockburnianus* Focke |  | X.R. Wang *R0255* | 2*x*^b^ | Baotianman, He'nan, China | MF595610 |  |  |
| *R. idaeopsis* Focke |  | Y. Wang & L. Zhang *R2218* | 2*x*^a^ | Baotianman, He'nan, China | MF595624 |  |  |
| *R. idaeopsis* Focke |  | Y. Wang & L. Zhang *R2219* | 2*x*^a^ | Baotianman, He'nan, China | MF595625 |  |  |
| *R. innominatus* S. Moore |  | Y. Wang & L. Zhang *R2008* | 2*x*^a^ | Mt. Leigong, Guizhou, China | MF595627 |  |  |
| *R. innominatus* S. Moore var. *kuntzeanus* (Hemsl) |  | Y. Wang & L. Zhang *R2327* | 2*x*^a^ | Mt. Nangong, Shaanxi, China | MF595630 |  |  |
| *R. innominatus* S. Moore var. *macrosepalus* |  | Y. Wang & L. Zhang *R2313* | 2*x*^a^ | Mt. Tiantai, Shaanxi, China | MF595631 |  |  |
| *R. innominatus* S. Moore var. *macrosepalus* |  | Y. Wang & L. Zhang *R2328* | 2*x*^a^ | Mt. Nangong, Shaanxi, China | MF595632 |  |  |
| *R. innominatus* S. Moore var. *macrosepalus* |  | Y. Wang & L. Zhang *R2332* | 2*x*^a^ | Mt. Nangong, Shaanxi, China | MF595633 |  |  |
| *R. innominatus* S. Moore var. *quinatus* |  | Y. Wang & L. Zhang *R2107* | 2*x*^a^ | Mt. Lu, Jiangxi, China | MF595634 |  |  |
| *R. innominatus* S. Moore var. *aralioides* (Hance) Yü et Lu |  | Y. Wang & L. Zhang *R2133* | 2*x*^a^ | Mt. Wugong, Jiangxi, China | MF595628 |  |  |
| *R. innominatus* S. Moore var. *aralioides* (Hance) Yü et Lu |  | Y. Wang & L. Zhang *R2135* | 2*x*^a^ | Mt. Wugong, Jiangxi, China | MF595629 |  |  |
| **2. Subsect. *Idaeanthi* (Focke) Yü et Lu** | **12** |  |  |  |  |  |  |
| *R. niveus* Thunb. |  | X.R. Wang *R0101* | 2*x*^b^ | Mt. Zhougong, Sichuan, China | MF595645 |  |  |
| *R. piluliferus* Focke |  | Y. Wang & L. Zhang *R2204* | NA | Baotianman, He'nan, China | MF595660 |  |  |
| *R. piluliferus* Focke |  | Y. Wang & L. Zhang *R2303* | NA | Mt. Tiantai, Shaanxi, China | MF595661 |  |  |
| *R. piluliferus* Focke |  | Y. Wang & L. Zhang *R2406* | NA | Liangdang, Gansu, China | MF595662 |  |  |
| *R. piluliferus* Focke |  | Y. Wang & L. Zhang *R2407* | NA | Liangdang, Gansu, China | MF595663 |  |  |
| *R. eucalyptus* Focke |  | Y. Wang & L. Zhang *R2354* | NA | Qinling, Shaanxi, China | MF595619 |  |  |
| *R. idaeus* L. |  | L. Liu *R2516* | 2*x*^b^ | Motuo, Tibet, China | MF595626 |  |  |
| *R. aurantiacus* Focke |  | L. Liu *R2512* | NA | Lang County, Tibet, China | MF595605 |  |  |
| *R. austro-tibetanus* Yü et Lu |  | L. Liu *R2524* | NA | Milin, Tibet, China | MF595606 |  |  |
| *R. sachalinensis* Lévl. |  | Y. Wang & L. Zhang *R2215* | 4*x*^a^ | Baotianman, He'nan, China | MF595675 |  |  |
| *R. sachalinensis* Lévl. |  | Y. Wang & L. Zhang *R2220* | 4*x*^a^ | Baotianman, He'nan, China | MF595676 |  |  |
| *R. irritans* Focke |  | L. Liu *R2527* | NA | Linzhi, Tibet, China | MF595637 |  |  |
| **3. Subsect. *Pileati* Yü et Lu** | **4** |  |  |  |  |  |  |
| *R. pseudopileatus* Card. |  | X.R. Wang *R0238* | NA | Mt. E'mei, Sichuan, China | MF595667 |  |  |
| *R. subinopertus* Yü et Lu |  | X.R. Wang *R0207* | 2*x*^b^ | Mt. E'mei, Sichuan, China | MF595682 |  |  |
| *R. subinopertus* Yü et Lu |  | X.R. Wang *R0232* | 2*x*^b^ | Mt. E'mei, Sichuan, China | MF595683 |  |  |
| *R. pubifolius* Yü et Lu |  | L. Liu *R2536* | 2*x*^a^ | Yanjing, Tibet, China | MF595668 |  |  |
| **4. Subsect. *Stimulantes* Yü et Lu** | **22** |  |  |  |  |  |  |
| *R.* *phoenicolasius* Maxim. |  | Y. Wang & L. Zhang *R2351* | 2*x*^a^ | Qinling, Shaanxi, China | MF595659 |  |  |
| *R. ellipticus* Smith |  | X.R. Wang *R0112* | 2*x*^b^ | Mt. Zhougong, Sichuan, China |  | MF595617 |  |
| *R. ellipticus* Smith var. *obcordatus* (Franch) Focke |  | X.R. Wang *R0113* | 2*x*^b^ | Mt. Zhougong, Sichuan, China |  | MF595618 |  |
| *R. stimulans* Focke |  | Y. Wang & L. Zhang *R2210* | NA | Baotianman, He'nan, China | MF595680 |  |  |
| *R. stimulans* Focke |  | Y. Wang & L. Zhang *R2211* | NA | Baotianman, He'nan, China | MF595681 |  |  |
| *R. parvifolius* L. |  | X.R. Wang *R0140* | 3*x*^b^ | Yaan, Sichuan, China | MF595646 |  |  |
| *R. parvifolius* L. |  | X.R. Wang *R0343* | 2*x*^b^ | Xichong, Sichuan, China | MF595647 |  |  |
| *R. parvifolius* L. |  | X.R. Wang *R0345* | 2*x*^b^ | Xichong, Sichuan, China | MF595648 |  |  |
| *R. parvifolius* L. |  | X.R. Wang *R0349* | 2*x*^b^ | Xichong, Sichuan, China | MF595649 |  |  |
| *R. parvifolius* L. |  | Y. Wang & L. Zhang *R2101* | 2*x*^b^ | Luxi, Jiangxi, China | MF595650 |  |  |
| *R. parvifolius* L. |  | Y. Wang & L. Zhang *R2035* | 2*x*^b^ | Jiangkou, Guizhou, China | MF595651 |  |  |
| *R. parvifolius* L. |  | Y. Wang & L. Zhang *R2227* | 2*x*^b^ | Mt. Tongbai, He'nan, China | MF595652 |  |  |
| *R. kulinganus* Bailey |  | Y. Wang & L. Zhang *R2106* | NA | Mt. Lu, Jiangxi, China | MF595638 |  |  |
| *R. mesogaeus* Focke |  | X.R. Wang *R0107* | 2*x*^a^ | Mt. Zhougong, Sichuan, China | MF595642 |  |  |
| *R. mesogaeus* Focke |  | Y. Wang & L. Zhang *R2020* | 2*x*^a^ | Mt. Fanjing, Guizhou, China | MF595643 |  |  |
| *R. mesogaeus* Focke var. *oxycomus* Focke |  | X.R. Wang *R0150* | 2*x*^a^ | Mt. Zhougong, Sichuan, China | MF595644 |  |  |
| *R. subtibetanus* Hand.-Mazz. |  | Y. Wang & L. Zhang *R2027* | 2*x*^a^ | Mt. Fanjing, Guizhou, China | MF595684 |  |  |
| *R. subtibetanus* Hand.-Mazz. |  | Y. Wang & L. Zhang *R2302* | 2*x*^a^ | Mt. Tiantai, Shaanxi, China | MF595685 |  |  |
| *R. subtibetanus* Hand.-Mazz. |  | Y. Wang & L. Zhang *R2348* | 2*x*^a^ | Qinling, Shaanxi, China | MF595686 |  |  |
| *R. subtibetanus* Hand.-Mazz. var. *glandulosus* Yü et Lu |  | Y. Wang & L. Zhang *R2402* | 2*x*^a^ | Liangdang, Gansu, China | MF595687 |  |  |
| *R. biflorus* Buch.-Ham. ex Smith |  | L. Liu *R2504* | 2*x*^a^ | Chayu, Tibet, China | MF595607 |  |  |
| *R. alexeterius* Focke var. *acaenocalyx* (Hara) Yü et Lu |  | L. Liu *R2513* | 2*x*^a^ | Lang County, Tibet, China | MF595603 |  |  |
| **5. Subsect. *Pungentes* (Focke) Yü et Lu** | **17** |  |  |  |  |  |  |
| *R. inopertus* (Diels) Focke |  | X.R. Wang *R0105* | 2*x*^a^ | Mt. Zhougong, Sichuan, China | MF595635 |  |  |
| *R. inopertus* (Diels) Focke var. *echinocalyx* Card. |  | X.R. Wang *R0108* | 2*x*^a^ | Mt. Zhougong, Sichuan, China | MF595636 |  |  |
| *R. amabilis* Focke |  | X.R. Wang *R0227* | 2*x*^a^ | Mt. E'mei, Sichuan, China | MF595604 |  |  |
| *R. pinfaensis* Lévl. et Vant |  | X.R. Wang *R0102* | 2*x*^a^ | Yaan, Sichuan, China |  | MF595664 |  |
| *R. pinfaensis* Lévl. et Vant |  | X.R. Wang *R0103* | 2*x*^a^ | Yaan, Sichuan, China |  | MF595665 |  |
| *R. pinfaensis* Lévl. et Vant |  | X.R. Wang *R0271* | 2*x*^a^ | Mt. E'mei, Sichuan, China |  | MF595666 |  |
| *R. coreanus* Miq. |  | Y. Wang & L. Zhang *R2017* | 2*x*^b^ | Mt. Fanjing, Guizhou, China | MF595614 |  |  |
| *R. stans* Focke |  | L. Liu *R2528* | 2*x*^b^ | Linzhi, Tibet, China | MF595679 |  |  |
| *R. pungens* Camb. |  | Y. Wang & L. Zhang *R2337* | 2*x*^b^ | Qinling, Shaanxi, China | MF595669 |  |  |
| *R. pungens* Camb. var. *villosus* Card. |  | Y. Wang & L. Zhang *R2405* | 2*x*^b^ | Liangdang, Gansu, China | MF595673 |  |  |
| *R. pungens* Camb. var. *oldhamii* (Miq.) Maxim. |  | Y. Wang & L. Zhang *R2307* | 2*x*^b^ | Mt. Tiantai, Shaanxi, China | MF595671 |  |  |
| *R. pungens* Camb. var. *oldhamii* (Miq.) Maxim. |  | Y. Wang & L. Zhang *R2318* | 2*x*^b^ | Mt. Zibai, Shaanxi, China | MF595672 |  |  |
| *R. pungens* Camb. var. *linearisepalus* Yü et Lu |  | X.R. Wang *R0229* | 2*x*^b^ | Mt. E'mei, Sichuan, China | MF595670 |  |  |
| *R. macilentus* Camb. |  | L. Liu *R2501* | 2*x*^a^ | Xiachayu, Tibet, China |  | MF595640 |  |
| *R. macilentus* Camb. var. *angulatus* Delav. |  | X.R. Wang *R0247* | 2*x*^a^ | Mt. E'mei, Sichuan, China |  | MF595641 |  |
| *R. simplex* Focke |  | Y. Wang & L. Zhang *R2321* | 2*x*^a^ | Mt. Zibai, Shaanxi, China |  | MF595677 |  |
| *R. simplex* Focke |  | Y. Wang & L. Zhang *R2323* | 2*x*^a^ | Mt. Zibai, Shaanxi, China |  | MF595678 |  |
| **6. Subsect. *Rosaefolii* (Focke) Yü et Lu** | **6** |  |  |  |  |  |  |
| *R. sumatranus* Miq. |  | Y. Wang & L. Zhang *R2111* | 2*x*^a^ | Mt. Lu, Jiangxi, China |  | MF595688 |  |
| *R. tsangii* Merr. |  | X.R. Wang *R0103* | 2*x*^a^ | Mt. Zhougong, Sichuan, China |  | MF595689 |  |
| *R. tsangii* Merr. var. *linearifoliolus* (Hayata) Yü et Lu |  | Y. Wang & L. Zhang *R2131* | 2*x*^a^ | Mt. Wugong, Jiangxi, China |  | MF595690 |  |
| *R. rosaefolius* Smith |  | Y. Wang & L. Zhang *R2005* | NA | Mt. Leigong, Guizhou, China |  | MF595674 |  |
| *R. eustephanus* Focke ex Diels var. *glanduliger* Yü et Lu |  | L. Liu *R2518* | 2*x*^a^ | Motuo, Tibet, China |  | MF595620 |  |
| *R. hirsutus* Thunb. |  | Y. Wang & L. Zhang *R2225* | 2*x*^a^ | Mt. Tongbai, He'nan, China |  | MF595623 |  |
| **7. Subsect. *Leucanthi* (Focke) Yü et Lu** | **1** |  |  |  |  |  |  |
| *R. columellaris* Tutcher |  | Y. Wang & L. Zhang *R2002* | 2*x*^a^ | Mt. Leigong, Guizhou, China |  | MF595611 |  |
| **8. Subsect. *Wushanenses* Yü et Lu** | **1** |  |  |  |  |  |  |
| *R. lasiostylus* Focke |  | Y. Wang & L. Zhang *R2339* | 2*x*^a^ | Qinling, Shaanxi, China | MF595639 |  |  |
| **9. Subsect. *Alpestres* (Focke) Yü et Lu** | **2** |  |  |  |  |  |  |
| *R. pentagonus* Wall. ex Focke |  | X.R. Wang *R0223* | 2*x*^a^ | Mt. E'mei, Sichuan, China | MF595655 | MF595656 |  |
| *R. pentagonus* Wall. ex Focke var. *modestus* Yü et Lu |  | X.R. Wang *R0206* | 2*x*^a^ | Mt. E'mei, Sichuan, China | MF595657 | MF595658 |  |
| **10. Subsect. *Peltati* (Focke) Yü et Lu** | **1** |  |  |  |  |  |  |
| *R. peltatus* Maxim. |  | Y. Wang & L. Zhang *R2012* | 2*x*^a^ | Mt. Leigong, Guizhou, China | MF595653 | MF595654 |  |
| **11. Subsect. *Corchorifolii* (Focke) Yü et Lu** | **7** |  |  |  |  |  |  |
| *R. corchorifolius* L. f. |  | X.R. Wang *R0115* | 2*x*^a^ | Mt. Zhougong, Sichuan, China | MF595612 |  |  |
| *R. corchorifolius* L. f. |  | Y. Wang & L. Zhang *R2032* | 2*x*^a^ | Jiangkou, Guizhou, China | MF595613 |  |  |
| *R. glabricarpus* Cheng |  | Y. Wang & L. Zhang *R2025* | NA | Mt. Fanjing, Guizhou, China | MF595621 |  |  |
| *R. grayanus* Maxim. |  | Y. Wang & L. Zhang *R2009* | 2*x*^c^ | Mt. Leigong, Guizhou, China |  | MF595622 |  |
| *R. crataegifolius* Bge. |  | Y. Wang & L. Zhang *R2016* | 2*x*^a^ | Mt. Leigong, Guizhou, China |  | MF595615 |  |
| *R. crataegifolius* Bge. |  | Y. Wang & L. Zhang *R2108* | 2*x*^a^ | Mt. Lu, Jiangxi, China |  | MF595616 |  |
| *R. chingii* Hu |  | Y. Wang & L. Zhang *R2128* | 2*x*^a^ | Mt. Wugong, Jiangxi, China | MF595609 |  |  |
| **Cultivar** | **1** |  |  |  |  |  |  |
| Raspberry cultivar ‘Chilcotin’ |  | X.R. Wang *R0139* | 2*x*^b^ | Introduced from Canada | MF595608 |  |  |
| **Sect. *Rubus*** | **1** |  |  |  |  |  |  |
| Blackberry cultivar ‘Arapaho’ |  | X.R. Wang *R0142* | 4*x*^b^ | Introduced from America | MF595696 |  |  |
| **Sect. *Malachobatus* Focke** | **47** |  |  |  |  |  |  |
| **16. Subsect. *Acuminati* (Focke) Yü et Lu** | **6** |  |  |  |  |  |  |
| *R. acuminatus* Smith var. *puberulus* Yü et Lu |  | Y. Wang & L. Zhang *R2007* | 4*x*^a^ | Mt. Leigong, Guizhou, China | MF595691 | MF595692 |  |
| *R. lambertianus* Ser. |  | Y. Wang & L. Zhang *R2018* | 4*x*^a^ | Mt. Fanjing, Guizhou, China | MF595735 | MF595736 | MF595737^#^ |
| *R. lambertianus* Ser. |  | Y. Wang & L. Zhang *R2304* | 4*x*^a^ | Mt. Tiantai, Shaanxi, China | MF595738 | MF595739^#^ | MF595740^#^ |
| *R. lambertianus* Ser. var. *glaber* Hemsl. |  | X.R. Wang *R0116* | 4*x*^a^ | Mt. Zhangjia, Sichuan, China | MF595741 | MF595742 |  |
| *R. lambertianus* Ser. var. *paykouangensis* |  | Y. Wang & L. Zhang *R2109* | 4*x*^a^ | Mt. Lu, Jiangxi, China | MF595743 |  | MF595744^#^ |
| *R. lambertianus* Ser. var. *paykouangensis* |  | Y. Wang & L. Zhang *R2121* | 4*x*^a^ | Mt. Wugong, Jiangxi, China | MF595745 |  | MF595746^#^ |
| **17. Subsect. *Dolichophylli* Yü et Lu** | **3** |  |  |  |  |  |  |
| *R. parkeri* Hance |  | X.R. Wang *R0117* | 4*x*^b^ | Mt. Zhangjia, Sichuan, China | MF595761 | MF595762 |  |
| *R. panduratus* Hand.- Mazz. |  | Y. Wang & L. Zhang *R2014* | NA | Mt. Leigong, Guizhou, China | MF595758 | MF595759 | MF595760^#^ |
| *R. ichangensis* Hemsl. et Ktze. |  | X.R. Wang *R0124* | 4*x*^b^ | Mt. Zhougong, Sichuan, China | MF595732 | * |  |
| **18. Subsect. *Elongati* (Focke) Yü et Lu** | **7** |  |  |  |  |  |  |
| *R. assamensis* Focke |  | X.R. Wang *R0118* | 4*x*^a^ | Mt. Zhangjia, Sichuan, China | MF595697 | MF595698 |  |
| *R. chroosepalus* Focke |  | X.R. Wang *R0237* | 4*x*^a^ | Mt. E'mei, Sichuan, China | MF595708 | MF595709 |  |
| *R. sempervirens* Yü et Lu |  | Y. Wang & L. Zhang *R2124* | NA | Mt. Wugong, Jiangxi, China | MF595777 | MF595778 |  |
| *R. feddei* Lévl. et Vant. |  | Y. Wang & L. Zhang *R2022* | 4*x*^a^ | Mt. Fanjing, Guizhou, China | MF595716 | MF595717 |  |
| *R. feddei* Lévl. et Vant. |  | Y. Wang & L. Zhang *R2031* | 4*x*^a^ | Mt. Fanjing, Guizhou, China | MF595718 | MF595719 |  |
| *R. tephrodes* Hance |  | Y. Wang & L. Zhang *R2034* | 4*x*^a^ | Jiangkou, Guizhou, China | MF595791 | MF595792 |  |
| *R. tephrodes* Hance var. *setosissimus* Hand.-Mazz. |  | Y. Wang & L. Zhang *R2117* | 4*x*^a^ | Luxi, Jiangxi, China | MF595793 | MF595794 |  |
| **19 Subsect. *Moluccani* (Focke) Yü et Lu** | **16** |  |  |  |  |  |  |
| *R. alceaefolius* Poir. |  | Y. Wang & L. Zhang *R2114* | 4*x*^d^ | Mt. Wugong, Jiangxi, China | * | MF595693 |  |
| *R. echinoides* Metc. |  | Y. Wang & L. Zhang *R2144* | 4*x*^a^ | Mt. Sanqing, Jiangxi, China | MF595710 | MF595711 |  |
| *R. rufus* Focke |  | X.R. Wang *R0111* | 4*x*^a^ | Mt. Zhougong, Sichuan, China | MF595769 | MF595770 |  |
| *R. rufus* Focke |  | X.R. Wang *R0123* | 4*x*^a^ | Mt. Zhougong, Sichuan, China | MF595771 | MF595772 |  |
| *R. rufus* Focke |  | X.R. Wang *R0146* | 4*x*^a^ | Mt. Zhougong, Sichuan, China | MF595773 | MF595774 |  |
| *R. rufus* Focke var. *palmatifidus* Card. |  | X.R. Wang *R0136* | 4*x*^a^ | Mt. Zhougong, Sichuan, China | MF595775 | MF595776 |  |
| *R. lasiotrichos* Focke |  | X.R. Wang *R0260* | NA | Mt. E'mei, Sichuan, China | MF595747 | MF595748 |  |
| *R. multibracteatus* Lévl. et Vant. |  | X.R. Wang *R0119* | 4*x*^a^ | Mt. Zhougong, Sichuan, China | MF595753 | MF595754 |  |
| *R. reticulatus* Wall. ex Hook. f. |  | L. Liu *R2521* | NA | Motuo, Tibet, China | MF595767 | MF595768 |  |
| *R. setchuenensis* Bureau et Franch. |  | X.R. Wang *R0104* | 4*x*^a^ | Mt. Zhougong, Sichuan, China | MF595779 | MF595780 |  |
| *R. faberi* Focke |  | X.R. Wang *R0243* | 4*x*^a^ | Mt. E'mei, Sichuan, China | MF595712 | MF595713 |  |
| *R. faberi* Focke |  | X.R. Wang *R0246* | 4*x*^a^ | Mt. E'mei, Sichuan, China | MF595714 | MF595715 |  |
| *R. pinnatisepalus* Hemsl. |  | Y. Wang & L. Zhang *R2026* | NA | Mt. Fanjing, Guizhou, China | MF595763 | MF595764 |  |
| *R. hunanensis* Hand. - Mazz. |  | Y. Wang & L. Zhang *R2030* | 4*x*^a^ | Mt. Leigong, Guizhou, China | MF595728 | MF595729 |  |
| *R. buergeri* Miq. |  | X.R. Wang *R0122* | 8*x*^b^ | Mt. Zhougong, Sichuan, China | * | MF595701 |  |
| *R. hypopitys* Focke var. *hanmiensis* Yü et Lu |  | L. Liu *R2533* | NA | Motuo, Tibet, China | MF595730 | MF595731 |  |
| **21. Subsect. *Stipulosi* Yü et Lu** | **3** |  |  |  |  |  |  |
| *R. stipulosus* Yü et Lu |  | X.R. Wang *R0154* | NA | Mt. Zhougong, Sichuan, China | MF595781 | MF595782 |  |
| *R. irenaeus* Focke |  | Y. Wang & L. Zhang *R2013* | 6*x*^e^ | Mt. Leigong, Guizhou, China | MF595733 | MF595734 |  |
| *R. pacificus* Hance |  | Y. Wang & L. Zhang *R2112* | NA | Mt. Lu, Jiangxi, China | MF595756 | MF595757 |  |
| **22. Subsect. *Sozostyli* (Focke) Yü et Lu** | **12** |  |  |  |  |  |  |
| *R. playfairianus* Hemsl. |  | X.R. Wang *R0110* | NA | Mt. Zhougong, Sichuan, China | MF595765 | MF595766 |  |
| *R. bambusarum* Focke |  | X.R. Wang *R0145* | 4*x*^a^ | Mt. Zhougong, Sichuan, China | MF595699 | MF595700 |  |
| *R. henryi* Hemsl. et. Ktze |  | X.R. Wang *R0151* | 4*x*^a^ | Mt. Zhougong, Sichuan, China | MF595724 | MF595725 |  |
| *R. huangpingensis* Yü et Lu |  | Y. Wang & L. Zhang *R2010* | NA | Mt. Leigong, Guizhou, China | MF595726 | MF595727 |  |
| *R. swinhoei* Hance |  | Y. Wang & L. Zhang *R2015* | 4*x*^a^ | Mt. Fanjing, Guizhou, China | MF595783 | MF595784 |  |
| *R. swinhoei* Hance |  | Y. Wang & L. Zhang *R2028* | 4*x*^a^ | Mt. Fanjing, Guizhou, China | MF595785 | MF595786 |  |
| *R. swinhoei* Hance |  | Y. Wang & L. Zhang *R2129* | 4*x*^a^ | Mt. Wugong, Jiangxi, China | MF595787 | MF595788 |  |
| *R. swinhoei* Hance |  | Y. Wang & L. Zhang *R2132* | 4*x*^a^ | Mt. Wugong, Jiangxi, China | MF595789 | MF595790 |  |
| *R. caudifolius* Yü et Lu |  | Y. Wang & L. Zhang *R2001* | 4*x*^a^ | Mt. Leigong, Guizhou, China | MF595704 | MF595705 |  |
| *R. caudifolius* Yü et Lu |  | Y. Wang & L. Zhang *R2021* | 4*x*^a^ | Mt. Fanjing, Guizhou, China | MF595706 | MF595707 |  |
| *R. malifolius* Focke |  | X.R. Wang *R0109* | NA | Mt. Zhougong, Sichuan, China | MF595749 | MF595750 |  |
| *R. malifolius* Focke |  | X.R. Wang *R0147* | NA | Mt. Zhougong, Sichuan, China | MF595751 | MF595752 |  |
| **Sect. *Dalibardastrum* (Focke) Yü et Lu** | **2** |  |  |  |  |  |  |
| *R. tsangorum* Hand.-Mazz. |  | Y. Wang & L. Zhang *R2142* | 4*x*^a^ | Mt. Sanqing, Jiangxi, China | MF595795 | MF595796 |  |
| *R. amphidasys* Focke ex Diels |  | Y. Wang & L. Zhang *R2115* | 6*x*^a^ | Mt. Lu, Jiangxi, China | MF595694 | MF595695 |  |
| **Sect. *Chamaebatus* Focke** | **1** |  |  |  |  |  |  |
| *R. calycinus* Wall. Ex D. Don |  | L. Liu *R2519* | 6*x*^a^ | Damu, Tibet, China | MF595702 | MF595703 |  |
| **Sect. *Cylactis* Focke** | **3** |  |  |  |  |  |  |
| *R. fockeanus* Kurz |  | L. Liu *R2523* | NA | Galongla, Tibet, China | MF595720 | MF595721 |  |
| *R. nyalamensis* Yü et Lu |  | L. Liu *R2534* | NA | Motuo, Tibet, China | * | MF595755 |  |
| *R. fragarioides Bertol.* var. *pubescens Franch* |  | L. Liu *R2530* | NA | Motuo, Tibet, China | MF595722^#^ | MF595723 |  |
| **Sect. *Anoplobatus* Focke** | **1** |  |  |  |  |  |  |
| *R. odoratus* L. |  | — | 2*x*^f^ | From GenBank | AF285994.1^g^ |  |  |
| **Outgroups** | **13** |  |  |  |  |  |  |
| **Maloideae** |  |  |  |  |  |  |  |
| *Amelanchier bartramiana* e |  | — | — | From GenBank | AF285976^g^ |  |  |
| *Amelanchier bartramiana* 7 |  | — | — | From GenBank | AF285975^g^ |  |  |
| *Amelanchier laevis* L19.1 |  | — | — | From GenBank | DQ874896^g^ |  |  |
| *Amelanchier laevis* L19.11 |  | — | — | From GenBank | DQ874897^g^ |  |  |
| *Amelanchier laevis* L19.12 |  | — | — | From GenBank | DQ874898^g^ |  |  |
| *Sorbus americana* c4 |  | — | — | From GenBank | AF500468^h^ |  |  |
| *Sorbus americana* c11 |  | — | — | From GenBank | AF500466^h^ |  |  |
| **Spiraeiudeae** |  |  |  |  |  |  |  |
| *Kageneckia oblonga* a |  | — | — | From GenBank | DQ874892^i^ |  |  |
| *Kageneckia oblonga* g |  | — | — | From GenBank | DQ874893^i^ |  |  |
| *Kageneckia oblonga* ca |  | — | — | From GenBank | AF285980^g^ |  |  |
| **Amygdloideae** |  |  |  |  |  |  |  |
| *Prinsepia sinensis* |  | — | — | From GenBank | AF285990^g^ |  |  |
| *Prunus virginiana* |  | — | — | From GenBank | AF500453^g^ |  |  |
| **Rosoideae** |  |  |  |  |  |  |  |
| *Rosa multiflora* |  | — | — | From GenBank | AF285993^g^ |  |  |

Note: ^a^Thompson (1997) [9]; ^b^Wang et al. (2008) [11]; ^c^Naruhashi et al. (2002) [10]; ^d^Amsellem et al. (2001) [50]; ^e^Meng & Finn (2002) [51]; ^f^Thompson (1995) [49]; ^g^Evans et al. (2000) [29]; ^h^Evans & Campbell (2002) [31]; ^i^Campbell et al. (2007) [54]; NA, not available.

^*^ indicates that the *GBSS*I-1a or *GBSS*I-1b homoeolog existed in the polyploids (PCR products) which failed to obtain the sequences.

^#^ represents that sequences contain stop codons and may become pseudogenes.
