# Supplementary material for: Allopolyploid origin in Rubus (Rosaceae) inferred from nuclear granule-bound starch synthase I (GBSSI) sequences
Source: BMC Plant Biol. 2019 Jul 10;19:303. doi: 10.1186/s12870-019-1915-7 (PMC6617891; doi:10.1186/s12870-019-1915-7)
Supplement: Supplementary file 3 — The identity and E-value in GBSSI-1 of Rubus species by alignment with reference genome of diploid R. occidentalis L. (DOCX 96 kb) [file 12870_2019_1915_MOESM3_ESM.docx]

**Additional file 3** The identity and e-value in *GBSS*I-1 of *Rubus* species by alignment with reference genome of diploid *R. occidentalis*

| ID1 | ID2^*^ | Identity | Alignment length | Mismatches | Gap openings | ID1 start | ID1 end | ID2 start | ID2 end | E-value | Bitscore |
| --- | --- | --- | --- | --- | --- | --- | --- | --- | --- | --- | --- |
| **Section *Idaeobatus*** |  |  |  |  |  |  |  |  |  |  |  |
| **1. Subsection *Thyrsidaei* (Focke) Yü et Lu** |  |  |  |  |  |  |  |  |  |  |  |
| *R. cockburnianus* R0255 | Ro07 | 97.15 | 1229 | 17 | 7 | 19 | 1230 | 892356 | 893583 | 0.00e^+00^ | 2044 |
| *R. idaeopsis* R2218 | Ro07 | 98.22 | 1238 | 10 | 5 | 10 | 1238 | 892349 | 893583 | 0 | 2153 |
| *R. idaeopsis* R2219 | Ro07 | 98.24 | 1249 | 9 | 6 | 4 | 1242 | 892350 | 893595 | 0 | 2167 |
| *R. innominatus* R2008 | Ro07 | 94.95 | 1228 | 45 | 3 | 18 | 1228 | 892356 | 893583 | 0 | 1794 |
| *R. innominatus* var. *kuntzeanus* R2327 | Ro07 | 97.08 | 1234 | 18 | 4 | 21 | 1236 | 892360 | 893593 | 0 | 2085 |
| *R. innominatus* var. *macrosepalus* R2313 | Ro07 | 96.86 | 1243 | 19 | 6 | 7 | 1229 | 892353 | 893595 | 0 | 2064 |
| *R. innominatus* var. *macrosepalus* R2328 | Ro07 | 97 | 1235 | 19 | 4 | 14 | 1230 | 892356 | 893590 | 0 | 2074 |
| *R. innominatus* var. *macrosepalus* R2332 | Ro07 | 96.85 | 1238 | 20 | 4 | 18 | 1236 | 892356 | 893593 | 0 | 2089 |
| *R. innominatus* var. *quinatus* R2107 | Ro07 | 96.89 | 1223 | 18 | 5 | 23 | 1225 | 892361 | 893583 | 0 | 2048 |
| *R. innominatus* var. *aralioides* R2133 | Ro07 | 96.81 | 1224 | 20 | 5 | 18 | 1222 | 892360 | 893583 | 0 | 2034 |
| *R. innominatus* var. *aralioides* R2135 | Ro07 | 97.08 | 1231 | 18 | 4 | 22 | 1234 | 892359 | 893589 | 0 | 2079 |
| **2. Subsect. *Idaeanthi* (Focke) Yü et Lu** |  |  |  |  |  |  |  |  |  |  |  |
| *R. niveus* R0101 | Ro07 | 96.97 | 1288 | 17 | 6 | 1 | 1268 | 892323 | 893608 | 0.00e^+00^ | 2127 |
| *R. piluliferus* R2204 | Ro07 | 96.7 | 1242 | 22 | 6 | 12 | 1234 | 892353 | 893594 | 0 | 2020 |
| *R. piluliferus* R2303 | Ro07 | 96.78 | 1243 | 21 | 6 | 5 | 1228 | 892353 | 893595 | 0 | 2030 |
| *R. piluliferus* R2406 | Ro07 | 96.83 | 1231 | 21 | 5 | 13 | 1225 | 892353 | 893583 | 0 | 2022 |
| *R. piluliferus* R2407 | Ro07 | 96.77 | 1237 | 21 | 6 | 18 | 1235 | 892356 | 893592 | 0 | 2018 |
| *R. eucalyptus* R2354 | Ro07 | 94.3 | 1246 | 31 | 6 | 20 | 1240 | 892359 | 893589 | 0.00e^+00^ | 1867 |
| *R. idaeus* R2516 | Ro07 | 96.52 | 1234 | 23 | 5 | 6 | 1222 | 892353 | 893583 | 0 | 1990 |
| *R. aurantiacus* R2512 | Ro07 | 92.28 | 1257 | 44 | 11 | 14 | 1235 | 892357 | 893595 | 0.00e^+00^ | 1665 |
| *R. austro-tibetanus* R2524 | Ro07 | 98.23 | 1241 | 12 | 4 | 13 | 1245 | 892357 | 893595 | 0.00e^+00^ | 2161 |
| *R. sachalinensis* R2215 | Ro07 | 94.13 | 954 | 30 | 7 | 14 | 944 | 892361 | 893311 | 0 | 1394 |
| *R. sachalinensis* R2215 | Ro07 | 96.41 | 251 | 8 | 1 | 967 | 1216 | 893345 | 893595 | 3.00e^-115^ | 418 |
| *R. sachalinensis* R2220 | Ro07 | 95.77 | 1228 | 37 | 3 | 21 | 1233 | 892356 | 893583 | 0 | 1909 |
| *R. irritans* R2527 | Ro07 | 91.97 | 1258 | 47 | 12 | 13 | 1235 | 892357 | 893595 | 0.00e^+00^ | 1628 |
| **3. Subsect. *Pileati* Yü et Lu** |  |  |  |  |  |  |  |  |  |  |  |
| *R. pseudopilearus* R0238 | Ro07 | 98.29 | 1228 | 10 | 4 | 12 | 1229 | 892357 | 893583 | 0 | 2149 |
| *R. subinopertus* R0207 | Ro07 | 96.61 | 1238 | 19 | 7 | 12 | 1229 | 892349 | 893583 | 0 | 1996 |
| *R. subinopertus* R0232 | Ro07 | 96.77 | 1240 | 17 | 7 | 11 | 1229 | 892357 | 893594 | 0 | 2016 |
| *R. pubifolius* R2536 | Ro07 | 97.92 | 1249 | 10 | 6 | 42 | 1275 | 892361 | 893608 | 0 | 2141 |
| **4. Subsect. *Stimulantes* Yü et Lu** |  |  |  |  |  |  |  |  |  |  |  |
| *R. phoenicolasius* R2351 | Ro07 | 96.77 | 1238 | 23 | 5 | 21 | 1241 | 892356 | 893593 | 0 | 2024 |
| *R. ellipticus* R0112 | Ro07 | 94.69 | 433 | 17 | 4 | 15 | 445 | 892323 | 892751 | 0 | 648 |
| *R. ellipticus* R0112 | Ro07 | 93.09 | 608 | 24 | 6 | 442 | 1046 | 892990 | 893582 | 0.00e^+00^ | 767 |
| *R. ellipticus* var. *obcordatus* R0113 | Ro07 | 94.75 | 400 | 16 | 3 | 23 | 421 | 892356 | 892751 | 5.00e^-172^ | 607 |
| *R. ellipticus* var. *obcordatus* R0113 | Ro07 | 93.07 | 649 | 25 | 8 | 418 | 1062 | 892990 | 893622 | 0 | 809 |
| *R. stimulans* R2210 | Ro07 | 96.77 | 1239 | 21 | 6 | 20 | 1239 | 892357 | 893595 | 0 | 2022 |
| *R. stimulans* R2211 | Ro07 | 96.7 | 1243 | 22 | 6 | 6 | 1229 | 892353 | 893595 | 0 | 2022 |
| *R. parvifolius* R0140 | Ro07 | 96.86 | 1243 | 20 | 5 | 6 | 1229 | 892353 | 893595 | 0 | 2066 |
| *R. parvifolius* R0343 | Ro07 | 97.06 | 1223 | 19 | 3 | 11 | 1216 | 892360 | 893582 | 0 | 2066 |
| *R. parvifolius* R0345 | Ro07 | 96.98 | 1227 | 19 | 4 | 27 | 1235 | 892361 | 893587 | 0 | 2058 |
| *R. parvifolius* R0349 | Ro07 | 96.98 | 1225 | 18 | 5 | 26 | 1232 | 892358 | 893581 | 0 | 2046 |
| *R. parvifolius* R2035 | Ro07 | 96.66 | 1228 | 21 | 6 | 20 | 1227 | 892356 | 893583 | 0 | 2012 |
| *R. parvifolius* R2101 | Ro07 | 96.66 | 1229 | 23 | 4 | 15 | 1226 | 892356 | 893583 | 0 | 2036 |
| *R. parvifolius* R2227 | Ro07 | 96.6 | 1234 | 22 | 6 | 24 | 1240 | 892360 | 893590 | 0 | 2016 |
| *R. kulinganus* R2106 | Ro07 | 96.61 | 1238 | 21 | 8 | 18 | 1234 | 892356 | 893593 | 0 | 1986 |
| *R. mesogaeus* R0107 | Ro07 | 97.96 | 1227 | 14 | 4 | 8 | 1226 | 892359 | 893582 | 0.00e^+00^ | 2103 |
| *R. mesogaeus* R2020 | Ro07 | 97.84 | 1249 | 16 | 4 | 34 | 1272 | 892361 | 893608 | 0 | 2143 |
| *R. mesogaeus* var. *oxycomus* R0150 | Ro07 | 97.83 | 1247 | 16 | 5 | 4 | 1240 | 892350 | 893595 | 0 | 2123 |
| *R. subtibetanus* R2027 | Ro07 | 98.05 | 1232 | 11 | 6 | 22 | 1244 | 892356 | 893583 | 0 | 2117 |
| *R. subtibetanus* R2302 | Ro07 | 98.16 | 1250 | 12 | 5 | 7 | 1246 | 892352 | 893600 | 0 | 2161 |
| *R. subtibetanus* R2348 | Ro07 | 97.81 | 1233 | 12 | 9 | 14 | 1233 | 892353 | 893583 | 0 | 2058 |
| *R. subtibetanus* var. *glandulosus* R2402 | Ro07 | 98.14 | 1288 | 13 | 5 | 3 | 1280 | 892322 | 893608 | 0 | 2222 |
| *R. biflorus* R2504 | Ro07 | 96.39 | 1246 | 23 | 7 | 5 | 1231 | 892353 | 893595 | 0.00e^+00^ | 1982 |
| *R. alexeterius* var. *acaenocalyx* R2513 | Ro07 | 98.15 | 1240 | 13 | 4 | 6 | 1236 | 892350 | 893588 | 0.00e^+00^ | 2149 |
| **5. Subsect. *Pungentes* (Focke) Yü et Lu** |  |  |  |  |  |  |  |  |  |  |  |
| *R. inopertus* R0105 | Ro07 | 96.66 | 1228 | 18 | 6 | 26 | 1232 | 892358 | 893583 | 0 | 2000 |
| *R. inopertus* var. *echinocalyx* R0108 | Ro07 | 96.77 | 1237 | 18 | 6 | 24 | 1240 | 892356 | 893590 | 0 | 2018 |
| *R. amabilis* R0227 | Ro07 | 97.82 | 1238 | 15 | 6 | 13 | 1238 | 892357 | 893594 | 0.00e^+00^ | 2133 |
| *R. pinfaensis* R0135 | Ro07 | 95.31 | 405 | 15 | 2 | 5 | 408 | 892350 | 892751 | 0 | 640 |
| *R. pinfaensis* R0135 | Ro07 | 93.72 | 605 | 25 | 4 | 405 | 1007 | 892990 | 893583 | 0 | 803 |
| *R. pinfaensis* R0249 | Ro07 | 95.31 | 405 | 15 | 2 | 6 | 409 | 892350 | 892751 | 0 | 640 |
| *R. pinfaensis* R0249 | Ro07 | 94.16 | 616 | 23 | 4 | 406 | 1019 | 892990 | 893594 | 0 | 841 |
| *R. pinfaensis* R0271 | Ro07 | 95.32 | 427 | 15 | 3 | 23 | 447 | 892328 | 892751 | 0 | 668 |
| *R. pinfaensis* R0271 | Ro07 | 93.97 | 630 | 25 | 4 | 444 | 1071 | 892990 | 893608 | 0 | 852 |
| *R. coreanus* R2017 | Ro07 | 95.31 | 959 | 21 | 6 | 11 | 945 | 892353 | 893311 | 0.00e^+00^ | 1507 |
| *R. coreanus* R2017 | Ro07 | 97.6 | 250 | 6 | 0 | 968 | 1217 | 893345 | 893594 | 3.00e^-124^ | 448 |
| *R. stans* R2528 | Ro07 | 91.96 | 1257 | 47 | 12 | 12 | 1233 | 892357 | 893594 | 0 | 1626 |
| *R. pungens* R2337 | Ro07 | 98.13 | 1233 | 14 | 3 | 13 | 1237 | 892352 | 893583 | 0 | 2173 |
| *R. pungens* var. *villosus* R2405 | Ro07 | 97.98 | 1239 | 16 | 3 | 13 | 1243 | 892352 | 893589 | 0 | 2169 |
| *R. pungens* var. *oldhamii* R2307 | Ro07 | 97.99 | 1246 | 15 | 4 | 4 | 1240 | 892350 | 893594 | 0 | 2175 |
| *R. pungens* var. *oldhamii* R2318 | Ro07 | 97.99 | 1243 | 15 | 4 | 13 | 1246 | 892352 | 893593 | 0 | 2169 |
| *R. pungens* var. *linearisepalus* R0229 | Ro07 | 97.82 | 1241 | 16 | 5 | 16 | 1247 | 892352 | 893590 | 0 | 2141 |
| *R. macilentus* R2501 | Ro07 | 94.57 | 258 | 6 | 3 | 8 | 257 | 892357 | 892614 | 9.00e^-106^ | 387 |
| *R. macilentus* R2501 | Ro07 | 98.53 | 68 | 1 | 0 | 283 | 350 | 892684 | 892751 | 1.00e^-27^ | 127 |
| *R. macilentus* R2501 | Ro07 | 92.44 | 635 | 34 | 5 | 347 | 979 | 892990 | 893612 | 0.00e^+00^ | 775 |
| *R. macilentus* var. *angulatus* R0247 | Ro07 | 94.22 | 277 | 6 | 5 | 25 | 291 | 892338 | 892614 | 1.00e^-107^ | 392 |
| *R. macilentus* var. *angulatus* R0247 | Ro07 | 98.53 | 68 | 1 | 0 | 317 | 384 | 892684 | 892751 | 1.00e^-27^ | 127 |
| *R. macilentus* var. *angulatus* R0247 | Ro07 | 92.41 | 606 | 33 | 4 | 381 | 984 | 892990 | 893584 | 0.00e^+00^ | 741 |
| *R. simplex* R2321 | Ro07 | 95.73 | 281 | 4 | 3 | 8 | 280 | 892334 | 892614 | 3.00e^-124^ | 448 |
| *R. simplex* R2321 | Ro07 | 95.59 | 68 | 3 | 0 | 306 | 373 | 892684 | 892751 | 8.00e^-23^ | 111 |
| *R. simplex* R2321 | Ro07 | 93.49 | 630 | 28 | 4 | 370 | 997 | 892990 | 893608 | 0 | 829 |
| *R. simplex* R2323 | Ro07 | 95.72 | 257 | 3 | 3 | 20 | 268 | 892358 | 892614 | 2.00e^-112^ | 408 |
| *R. simplex* R2323 | Ro07 | 95.59 | 68 | 3 | 0 | 294 | 361 | 892684 | 892751 | 7.00e^-23^ | 111 |
| *R. simplex* R2323 | Ro07 | 92.72 | 604 | 29 | 5 | 358 | 958 | 892990 | 893581 | 0 | 747 |
| **6. Subsect. *Rosaefolii* (Focke) Yü et Lu** |  |  |  |  |  |  |  |  |  |  |  |
| *R. sumatranus* R2111 | Ro07 | 92.99 | 314 | 18 | 2 | 28 | 340 | 892339 | 892649 | 1.00e^-120^ | 436 |
| *R. sumatranus* R2111 | Ro07 | 95.83 | 72 | 3 | 0 | 340 | 411 | 892680 | 892751 | 3.00e^-25^ | 119 |
| *R. sumatranus* R2111 | Ro07 | 94.06 | 623 | 23 | 5 | 408 | 1027 | 892990 | 893601 | 0 | 872 |
| *R. tsangii* R0103 | Ro07 | 92.42 | 396 | 18 | 6 | 24 | 408 | 892357 | 892751 | 2.00e^-143^ | 511 |
| *R. tsangii* R0103 | Ro07 | 93.96 | 646 | 25 | 5 | 405 | 1048 | 892990 | 893623 | 0 | 902 |
| *R. tsangii* var. *linearifoliolus* R2131 | Ro07 | 90.91 | 396 | 25 | 4 | 21 | 406 | 892357 | 892751 | 2.00e^-134^ | 482 |
| *R. tsangii* var. *linearifoliolus* R2131 | Ro07 | 93.47 | 628 | 28 | 4 | 403 | 1030 | 892990 | 893604 | 0 | 864 |
| *R. rosaefolius* R2005 | Ro07 | 92.02 | 426 | 18 | 8 | 2 | 413 | 892328 | 892751 | 4.00e^-148^ | 527 |
| *R. rosaefolius* R2005 | Ro07 | 93.82 | 631 | 26 | 4 | 410 | 1039 | 892990 | 893608 | 0 | 880 |
| *R. eustephanus* var. *glanduliger* R2518 | Ro07 | 92.12 | 406 | 17 | 7 | 2 | 394 | 892348 | 892751 | 2.00e^-143^ | 511 |
| *R. eustephanus* var. *glanduliger* R2518 | Ro07 | 93.66 | 615 | 26 | 4 | 391 | 1004 | 892990 | 893592 | 0 | 848 |
| *R. hirsutus* R2225 | Ro07 | 91.87 | 406 | 18 | 7 | 11 | 404 | 892349 | 892751 | 6.00e^-141^ | 504 |
| *R. hirsutus* R2225 | Ro07 | 94.06 | 606 | 23 | 4 | 401 | 1005 | 892990 | 893583 | 0.00e^+00^ | 854 |
| **7. Subsect. *Leucanthi* (Focke) Yü et Lu** |  |  |  |  |  |  |  |  |  |  |  |
| *R. columellaris* R2002 | Ro07 | 96.81 | 251 | 6 | 2 | 29 | 277 | 892341 | 892591 | 3.00e^-115^ | 418 |
| *R. columellaris* R2002 | Ro07 | 92.64 | 611 | 27 | 5 | 387 | 996 | 892990 | 893583 | 0 | 759 |
| **8. Subsect. *Wushanenses* Yü et Lu** |  |  |  |  |  |  |  |  |  |  |  |
| *R. lasiostylus* R2339 | Ro07 | 97.89 | 1232 | 11 | 9 | 18 | 1236 | 892354 | 893583 | 0.00e^+00^ | 2064 |
| **9. Subsect. *Alpestres* (Focke) Yü et Lu** |  |  |  |  |  |  |  |  |  |  |  |
| *R. pentagonus* R0223a | Ro07 | 95.23 | 1259 | 41 | 6 | 19 | 1263 | 892341 | 893594 | 0 | 1939 |
| *R. pentagonus* R0223b | Ro07 | 95.29 | 403 | 15 | 2 | 9 | 410 | 892352 | 892751 | 0 | 636 |
| *R. pentagonus* R0223b | Ro07 | 93.25 | 607 | 26 | 5 | 407 | 1011 | 892990 | 893583 | 0 | 825 |
| *R. pentagonus* var. *modestus* R0206a | Ro07 | 95.23 | 1259 | 41 | 6 | 19 | 1263 | 892341 | 893594 | 0 | 1939 |
| *R. pentagonus* var. *modestus* R0206b | Ro07 | 95.29 | 403 | 15 | 2 | 9 | 410 | 892352 | 892751 | 0 | 636 |
| *R. pentagonus* var. *modestus* R0206b | Ro07 | 93.2 | 618 | 26 | 6 | 407 | 1021 | 892990 | 893594 | 0 | 831 |
| **10. Subsect. *Peltati* (Focke) Yü et Lu** |  |  |  |  |  |  |  |  |  |  |  |
| *R. peltatus* R2012a | Ro07 | 95.57 | 1286 | 30 | 9 | 1 | 1272 | 892324 | 893596 | 0 | 2002 |
| *R. peltatus* R2012b | Ro07 | 92.59 | 405 | 26 | 2 | 13 | 416 | 892350 | 892751 | 6.00e^-144^ | 513 |
| *R. peltatus* R2012b | Ro07 | 93.03 | 617 | 27 | 6 | 413 | 1024 | 892990 | 893595 | 0 | 821 |
| **11. Subsect. *Corchorifolii* (Focke) Yü et Lu** |  |  |  |  |  |  |  |  |  |  |  |
| *R. corchorifolius* R0115 | Ro07 | 92.72 | 1236 | 55 | 10 | 12 | 1218 | 892361 | 893590 | 0.00e^+00^ | 1659 |
| *R. corchorifolius* R2032 | Ro07 | 92.86 | 1247 | 54 | 10 | 5 | 1222 | 892350 | 893590 | 0.00e^+00^ | 1689 |
| *R. glabricarpus* R2025 | Ro07 | 92.34 | 1267 | 62 | 10 | 11 | 1248 | 892361 | 893621 | 0.00e^+00^ | 1665 |
| *R. grayanus* R2009 | Ro07 | 91.67 | 288 | 20 | 2 | 13 | 299 | 892352 | 892636 | 2.00e^-100^ | 369 |
| *R. grayanus* R2009 | Ro07 | 97.22 | 72 | 2 | 0 | 295 | 366 | 892680 | 892751 | 1.00e^-27^ | 127 |
| *R. grayanus* R2009 | Ro07 | 94.21 | 605 | 22 | 4 | 363 | 965 | 892990 | 893583 | 0.00e^+00^ | 827 |
| *R. crataegifolius* R2016 | Ro07 | 92.08 | 303 | 19 | 3 | 27 | 327 | 892337 | 892636 | 6.00e^-107^ | 391 |
| *R. crataegifolius* R2016 | Ro07 | 94.44 | 630 | 22 | 4 | 391 | 1018 | 892990 | 893608 | 0.00e^+00^ | 876 |
| *R. crataegifolius* R2108 | Ro07 | 92.41 | 290 | 18 | 2 | 4 | 292 | 892350 | 892636 | 2.00e^-106^ | 389 |
| *R. crataegifolius* R2108 | Ro07 | 94.17 | 617 | 22 | 5 | 356 | 969 | 892990 | 893595 | 0.00e^+00^ | 835 |
| *R. chingii* R2128 | Ro07 | 92.4 | 1250 | 55 | 9 | 12 | 1237 | 892350 | 893583 | 0.00e^+00^ | 1667 |
| **Cultivar** |  |  |  |  |  |  |  |  |  |  |  |
| Chilcotin R0139 | Ro07 | 99.51 | 1233 | 2 | 3 | 15 | 1245 | 892353 | 893583 | 0.00e^+00^ | 2298 |
| **Sect. *Rubus*** |  |  |  |  |  |  |  |  |  |  |  |
| Arapoho R0142a | Ro07 | 93.16 | 307 | 13 | 2 | 6 | 304 | 892351 | 892657 | 3.00e^-121^ | 438 |
| Arapoho R0142a | Ro07 | 98.77 | 489 | 5 | 1 | 407 | 894 | 892823 | 893311 | 0.00e^+00^ | 914 |
| Arapoho R0142a | Ro07 | 97.79 | 272 | 6 | 0 | 917 | 1188 | 893345 | 893616 | 2.00e^-137^ | 492 |
| **Sect. *Malachobatus* Focke** |  |  |  |  |  |  |  |  |  |  |  |
| **16. Subsect. *Acuminati* (Focke) Yü et Lu** |  |  |  |  |  |  |  |  |  |  |  |
| *R. acuminatus* var. *puberulus* R2007a | Ro07 | 95.62 | 1302 | 33 | 7 | 1 | 1291 | 892324 | 893612 | 0.00e^+00^ | 2042 |
| *R. acuminatus* var. *puberulus* R2007b | Ro07 | 92.84 | 405 | 24 | 3 | 6 | 408 | 892350 | 892751 | 7.00e^-156^ | 553 |
| *R. acuminatus* var. *puberulus* R2007b | Ro07 | 93.02 | 616 | 25 | 8 | 405 | 1013 | 892990 | 893594 | 0 | 803 |
| *R. lambertianus* R2018a | Ro07 | 95.36 | 1316 | 35 | 9 | 12 | 1315 | 892322 | 893623 | 0 | 2010 |
| *R. lambertianus* R2018b | Ro07 | 92.35 | 405 | 27 | 2 | 6 | 409 | 892350 | 892751 | 2.00e^-153^ | 545 |
| *R. lambertianus* R2018b | Ro07 | 92.86 | 616 | 27 | 7 | 406 | 1015 | 892990 | 893594 | 0 | 803 |
| *R. lambertianus* R2018c | Ro07 | 92.04 | 402 | 28 | 2 | 5 | 405 | 892353 | 892751 | 2.00e^-149^ | 531 |
| *R. lambertianus* R2018c | Ro07 | 96.3 | 81 | 3 | 0 | 402 | 482 | 892990 | 893070 | 1.00e^-30^ | 137 |
| *R. lambertianus* R2018c | Ro07 | 94.46 | 361 | 16 | 3 | 483 | 840 | 893235 | 893594 | 1.00e^-150^ | 535 |
| *R. lambertianus* R2304a | Ro07 | 94.62 | 1320 | 26 | 8 | 252 | 1539 | 892313 | 893619 | 0.00e^+00^ | 2010 |
| *R. lambertianus* R2304b | Ro07 | 92.59 | 405 | 26 | 2 | 7 | 410 | 892350 | 892751 | 7.00e^-156^ | 553 |
| *R. lambertianus* R2304b | Ro07 | 91.83 | 624 | 26 | 7 | 407 | 1024 | 892990 | 893594 | 0.00e^+00^ | 813 |
| *R. lambertianus* R2304c | Ro07 | 92.34 | 418 | 28 | 2 | 92 | 508 | 892337 | 892751 | 7.00e^-159^ | 563 |
| *R. lambertianus* R2304c | Ro07 | 96.3 | 81 | 3 | 0 | 505 | 585 | 892990 | 893070 | 1.00e^-30^ | 137 |
| *R. lambertianus* R2304c | Ro07 | 94.2 | 379 | 19 | 2 | 586 | 962 | 893235 | 893612 | 7.00e^-159^ | 563 |
| *R. lambertianus* var. *glaber* R0116a | Ro07 | 95.61 | 1254 | 28 | 8 | 3 | 1242 | 892350 | 893590 | 0.00e^+00^ | 1965 |
| *R. lambertianus* var. *glaber* R0116b | Ro07 | 92.12 | 406 | 27 | 3 | 7 | 411 | 892350 | 892751 | 2.00e^-149^ | 531 |
| *R. lambertianus* var. *glaber* R0116b | Ro07 | 93.34 | 616 | 25 | 6 | 408 | 1018 | 892990 | 893594 | 0 | 835 |
| *R. lambertianus* var. *paykouangensis* R2109a | Ro07 | 95.65 | 1287 | 29 | 9 | 1 | 1275 | 892323 | 893594 | 0.00e^+00^ | 1996 |
| *R. lambertianus* var. *paykouangensis* R2109c | Ro07 | 92.45 | 424 | 28 | 2 | 34 | 456 | 892331 | 892751 | 2.00e^-162^ | 575 |
| *R. lambertianus* var. *paykouangensis* R2109c | Ro07 | 96.3 | 81 | 3 | 0 | 453 | 533 | 892990 | 893070 | 1.00e^-30^ | 137 |
| *R. lambertianus* var. *paykouangensis* R2109c | Ro07 | 94.93 | 375 | 16 | 2 | 534 | 906 | 893235 | 893608 | 1.00e^-163^ | 579 |
| *R. lambertianus* var. *paykouangensis* R2121a | Ro07 | 95.72 | 1285 | 30 | 8 | 49 | 1322 | 892313 | 893583 | 0.00e^+00^ | 2006 |
| *R. lambertianus* var. *paykouangensis* R2121c | Ro07 | 92.18 | 409 | 28 | 2 | 11 | 418 | 892346 | 892751 | 1.00e^-153^ | 545 |
| *R. lambertianus* var. *paykouangensis* R2121c | Ro07 | 96.3 | 81 | 3 | 0 | 415 | 495 | 892990 | 893070 | 1.00e^-30^ | 137 |
| *R. lambertianus* var. *paykouangensis* R2121c | Ro07 | 94.93 | 375 | 16 | 2 | 496 | 868 | 893235 | 893608 | 1.00e^-163^ | 579 |
| **17. Subsect. *Dolichophylli* Yü et Lu** |  |  |  |  |  |  |  |  |  |  |  |
| *R. parkeri* R0117a | Ro07 | 95.22 | 481 | 8 | 3 | 14 | 486 | 892324 | 892797 | 0 | 771 |
| *R. parkeri* R0117a | Ro07 | 96.41 | 807 | 19 | 4 | 480 | 1283 | 892827 | 893626 | 0 | 1296 |
| *R. parkeri* R0117b | Ro07 | 93.22 | 398 | 23 | 2 | 26 | 422 | 892357 | 892751 | 7.00e^-159^ | 563 |
| *R. parkeri* R0117b | Ro07 | 93.03 | 617 | 26 | 7 | 419 | 1029 | 892990 | 893595 | 0 | 813 |
| *R. panduratus* R2014a | Ro07 | 94.93 | 1342 | 32 | 11 | 2 | 1329 | 892313 | 893632 | 0 | 2024 |
| *R. panduratus* R2014b | Ro07 | 93.56 | 404 | 22 | 2 | 10 | 412 | 892351 | 892751 | 8.00e^-165^ | 583 |
| *R. panduratus* R2014b | Ro07 | 93.1 | 623 | 26 | 7 | 409 | 1025 | 892990 | 893601 | 0 | 825 |
| *R. panduratus* R2014c | Ro07 | 93.54 | 418 | 23 | 2 | 152 | 568 | 892337 | 892751 | 8.00e^-171^ | 603 |
| *R. panduratus* R2014c | Ro07 | 93.88 | 147 | 6 | 1 | 565 | 711 | 892990 | 893133 | 3.00e^-44^ | 182 |
| *R. panduratus* R2014c | Ro07 | 96.4 | 222 | 7 | 1 | 735 | 956 | 893388 | 893608 | 2.00e^-100^ | 369 |
| *R. ichangensis* R0124a | Ro07 | 95.45 | 1297 | 32 | 8 | 3 | 1285 | 892349 | 893632 | 0.00e^+00^ | 2024 |
| **18. Subsect. *Elongati* (Focke) Yü et Lu** |  |  |  |  |  |  |  |  |  |  |  |
| *R. assamensis* R0118a | Ro07 | 95.55 | 494 | 6 | 4 | 10 | 495 | 892312 | 892797 | 0 | 797 |
| *R. assamensis* R0118a | Ro07 | 96.18 | 811 | 21 | 5 | 489 | 1294 | 892827 | 893632 | 0.00e^+00^ | 1255 |
| *R. assamensis* R0118b | Ro07 | 93.35 | 406 | 23 | 2 | 6 | 410 | 892349 | 892751 | 1.00e^-163^ | 579 |
| *R. assamensis* R0118b | Ro07 | 93.34 | 616 | 25 | 6 | 407 | 1017 | 892990 | 893594 | 0 | 835 |
| *R. chroosepalus* R0237a | Ro07 | 95.26 | 485 | 8 | 3 | 10 | 486 | 892320 | 892797 | 0 | 779 |
| *R. chroosepalus* R0237a | Ro07 | 96.45 | 760 | 18 | 4 | 480 | 1235 | 892827 | 893581 | 0 | 1195 |
| *R. chroosepalus* R0237b | Ro07 | 93.33 | 405 | 23 | 2 | 7 | 410 | 892350 | 892751 | 5.00e^-163^ | 577 |
| *R. chroosepalus* R0237b | Ro07 | 93.35 | 617 | 24 | 7 | 407 | 1017 | 892990 | 893595 | 0 | 829 |
| *R. sempervirens* R2124a | Ro07 | 97.73 | 44 | 1 | 0 | 13 | 56 | 892381 | 892338 | 4.00e^-13^ | 79.8 |
| *R. sempervirens* R2124a | Ro07 | 95.33 | 493 | 7 | 4 | 50 | 534 | 892313 | 892797 | 0 | 787 |
| *R. sempervirens* R2124a | Ro07 | 96.09 | 792 | 19 | 5 | 528 | 1312 | 892827 | 893613 | 0 | 1239 |
| *R. sempervirens* R2124b | Ro07 | 93.23 | 399 | 21 | 3 | 7 | 404 | 892350 | 892743 | 1.00e^-157^ | 559 |
| *R. sempervirens* R2124b | Ro07 | 93.22 | 605 | 25 | 6 | 409 | 1008 | 892990 | 893583 | 0 | 813 |
| *R. feddei* R2022a | Ro07 | 94.84 | 1260 | 37 | 9 | 2 | 1246 | 892348 | 893594 | 0.00e^+00^ | 1895 |
| *R. feddei* R2022b | Ro07 | 93.07 | 404 | 23 | 3 | 9 | 410 | 892351 | 892751 | 1.00e^-157^ | 559 |
| *R. feddei* R2022b | Ro07 | 93.18 | 616 | 25 | 7 | 407 | 1016 | 892990 | 893594 | 0 | 819 |
| *R. feddei* R2031a | Ro07 | 95.14 | 1277 | 33 | 10 | 5 | 1266 | 892350 | 893612 | 0.00e^+00^ | 1945 |
| *R. feddei* R2031b | Ro07 | 93.58 | 405 | 22 | 2 | 9 | 412 | 892350 | 892751 | 2.00e^-165^ | 585 |
| *R. feddei* R2031b | Ro07 | 93.34 | 616 | 24 | 7 | 409 | 1018 | 892990 | 893594 | 0 | 827 |
| *R. tephrodes* R2034a | Ro07 | 95.34 | 1287 | 33 | 8 | 41 | 1314 | 892352 | 893624 | 0 | 1996 |
| *R. tephrodes* R2034b | Ro07 | 93.09 | 405 | 24 | 2 | 6 | 409 | 892350 | 892751 | 1.00e^-160^ | 569 |
| *R. tephrodes* R2034b | Ro07 | 93.32 | 614 | 25 | 6 | 406 | 1014 | 892990 | 893592 | 0 | 831 |
| *R. tephrodes* var. *setosissimus* R2117a | Ro07 | 95.51 | 1336 | 31 | 10 | 49 | 1371 | 892313 | 893632 | 0 | 2077 |
| *R. tephrodes* var. *setosissimus* R2117b | Ro07 | 92.63 | 407 | 24 | 3 | 7 | 412 | 892350 | 892751 | 3.00e^-155^ | 551 |
| *R. tephrodes* var. *setosissimus* R2117b | Ro07 | 93.02 | 616 | 27 | 6 | 409 | 1019 | 892990 | 893594 | 0 | 819 |
| **19 Subsect. *Moluccani* (Focke) Yü et Lu** |  |  |  |  |  |  |  |  |  |  |  |
| *R. alceaefolius* R2114b | Ro07 | 92.33 | 404 | 27 | 2 | 9 | 411 | 892351 | 892751 | 6.00e^-141^ | 504 |
| *R. alceaefolius* R2114b | Ro07 | 93.02 | 616 | 26 | 7 | 408 | 1017 | 892990 | 893594 | 0.00e^+00^ | 811 |
| *R. echinoides* R2144a | Ro07 | 94.06 | 1313 | 51 | 9 | 4 | 1302 | 892324 | 893623 | 0.00e^+00^ | 1846 |
| *R. echinoides* R2144b | Ro07 | 92.57 | 404 | 26 | 2 | 8 | 410 | 892351 | 892751 | 2.00e^-143^ | 511 |
| *R. echinoides* R2144b | Ro07 | 93.02 | 616 | 26 | 7 | 407 | 1016 | 892990 | 893594 | 0 | 811 |
| *R. rufus* R0111a | Ro07 | 95.4 | 1325 | 34 | 8 | 13 | 1323 | 892321 | 893632 | 0 | 2064 |
| *R. rufus* R0111b | Ro07 | 92.48 | 399 | 26 | 2 | 16 | 413 | 892356 | 892751 | 3.00e^-152^ | 541 |
| *R. rufus* R0111b | Ro07 | 93.06 | 605 | 26 | 6 | 410 | 1009 | 892990 | 893583 | 0 | 805 |
| *R. rufus* R0123a | Ro07 | 95.22 | 1317 | 36 | 8 | 12 | 1314 | 892316 | 893619 | 0 | 2032 |
| *R. rufus* R0123b | Ro07 | 92.46 | 411 | 27 | 2 | 2 | 411 | 892344 | 892751 | 4.00e^-157^ | 557 |
| *R. rufus* R0123b | Ro07 | 93.02 | 616 | 27 | 6 | 408 | 1018 | 892990 | 893594 | 0 | 819 |
| *R. rufus* R0146a | Ro07 | 95.33 | 1306 | 34 | 8 | 21 | 1312 | 892324 | 893616 | 0 | 2026 |
| *R. rufus* R0146b | Ro07 | 92.48 | 399 | 26 | 2 | 16 | 413 | 892356 | 892751 | 3.00e^-152^ | 541 |
| *R. rufus* R0146b | Ro07 | 93.06 | 605 | 26 | 6 | 410 | 1009 | 892990 | 893583 | 0 | 805 |
| *R. rufus* var. *palmatifidus* R0136a | Ro07 | 95.16 | 1322 | 37 | 8 | 2 | 1309 | 892324 | 893632 | 0 | 2034 |
| *R. rufus* var. *palmatifidus* R0136b | Ro07 | 93.47 | 398 | 22 | 2 | 9 | 405 | 892350 | 892744 | 3.00e^-161^ | 571 |
| *R. rufus* var. *palmatifidus* R0136b | Ro07 | 93.19 | 617 | 25 | 7 | 409 | 1019 | 892990 | 893595 | 0 | 821 |
| *R. lasiotrichos* R0260a | Ro07 | 96.04 | 1312 | 27 | 6 | 305 | 1603 | 892313 | 893612 | 0 | 2119 |
| *R. lasiotrichos* R0260b | Ro07 | 94.33 | 406 | 18 | 3 | 7 | 411 | 892350 | 892751 | 8.00e^-171^ | 603 |
| *R. lasiotrichos* R0260b | Ro07 | 93.69 | 618 | 23 | 6 | 408 | 1022 | 892990 | 893594 | 0.00e^+00^ | 888 |
| *R. multibracteatus* R0119a | Ro07 | 95.74 | 1244 | 33 | 7 | 62 | 1292 | 892376 | 893612 | 0 | 1963 |
| *R. multibracteatus* R0119b | Ro07 | 93.12 | 407 | 24 | 2 | 4 | 409 | 892348 | 892751 | 7.00e^-162^ | 573 |
| *R. multibracteatus* R0119b | Ro07 | 93.18 | 616 | 26 | 6 | 406 | 1016 | 892990 | 893594 | 0 | 827 |
| *R. reticulatus* R2521a | Ro07 | 94.52 | 511 | 11 | 5 | 1 | 502 | 892352 | 892854 | 0 | 749 |
| *R. reticulatus* R2521a | Ro07 | 92 | 75 | 6 | 0 | 552 | 626 | 892855 | 892929 | 9.00e^-20^ | 101 |
| *R. reticulatus* R2521a | Ro07 | 95.83 | 648 | 16 | 4 | 625 | 1266 | 892953 | 893595 | 0 | 999 |
| *R. reticulatus* R2521b | Ro07 | 93.07 | 404 | 24 | 2 | 11 | 413 | 892351 | 892751 | 4.00e^-160^ | 567 |
| *R. reticulatus* R2521b | Ro07 | 93.51 | 616 | 24 | 6 | 410 | 1020 | 892990 | 893594 | 0 | 842 |
| *R. setchuenensis* R0106a | Ro07 | 96.02 | 1280 | 26 | 8 | 2 | 1265 | 892324 | 893594 | 0 | 2044 |
| *R. setchuenensis* R0106b | Ro07 | 94.54 | 403 | 17 | 3 | 10 | 410 | 892352 | 892751 | 2.00e^-171^ | 605 |
| *R. setchuenensis* R0106b | Ro07 | 93.85 | 618 | 23 | 5 | 407 | 1022 | 892990 | 893594 | 0 | 904 |
| *R. faberi* R0243a | Ro07 | 96.11 | 1312 | 26 | 6 | 313 | 1611 | 892313 | 893612 | 0.00e^+00^ | 2127 |
| *R. faberi* R0243b | Ro07 | 94.54 | 403 | 18 | 2 | 10 | 411 | 892352 | 892751 | 8.00e^-174^ | 613 |
| *R. faberi* R0243b | Ro07 | 93.21 | 619 | 26 | 6 | 408 | 1023 | 892990 | 893595 | 0.00e^+00^ | 866 |
| *R. faberi* R0246a | Ro07 | 96.25 | 1305 | 24 | 6 | 1 | 1292 | 892324 | 893616 | 0.00e^+00^ | 2129 |
| *R. faberi* R0246b | Ro07 | 94.54 | 403 | 18 | 2 | 9 | 410 | 892352 | 892751 | 8.00e^-174^ | 613 |
| *R. faberi* R0246b | Ro07 | 93.37 | 618 | 24 | 7 | 407 | 1020 | 892990 | 893594 | 0 | 864 |
| *R. pinnatisepalus* R2026a | Ro07 | 96.12 | 1313 | 25 | 7 | 2 | 1301 | 892313 | 893612 | 0 | 2121 |
| *R. pinnatisepalus* R2026b | Ro07 | 94.95 | 396 | 16 | 2 | 18 | 412 | 892359 | 892751 | 2.00e^-174^ | 615 |
| *R. pinnatisepalus* R2026b | Ro07 | 93.2 | 618 | 25 | 7 | 409 | 1022 | 892990 | 893594 | 0 | 856 |
| *R. hunanensis* R2030a | Ro07 | 95.38 | 1255 | 31 | 9 | 5 | 1245 | 892353 | 893594 | 0.00e^+00^ | 1933 |
| *R. hunanensis* R2030b | Ro07 | 92.35 | 405 | 27 | 2 | 10 | 413 | 892350 | 892751 | 1.00e^-141^ | 505 |
| *R. hunanensis* R2030b | Ro07 | 92.86 | 616 | 27 | 7 | 410 | 1019 | 892990 | 893594 | 0 | 803 |
| *R. buergeri* R0122b | Ro07 | 92.84 | 405 | 25 | 2 | 12 | 415 | 892350 | 892751 | 2.00e^-146^ | 521 |
| *R. buergeri* R0122b | Ro07 | 93.06 | 605 | 26 | 6 | 412 | 1011 | 892990 | 893583 | 0 | 805 |
| *R. hypopity* var. *shanmiensis* R2533a | Ro07 | 95.82 | 1315 | 27 | 8 | 3 | 1302 | 892322 | 893623 | 0.00e^+00^ | 2087 |
| *R. hypopity* var. *shanmiensis* R2533b | Ro07 | 94.81 | 405 | 17 | 2 | 9 | 412 | 892350 | 892751 | 2.00e^-177^ | 625 |
| *R. hypopity* var. *shanmiensis* R2533b | Ro07 | 93.64 | 613 | 27 | 6 | 409 | 1018 | 892990 | 893593 | 0 | 871 |
| **21. Subsect. *Stipulosi* Yü et Lu** |  |  |  |  |  |  |  |  |  |  |  |
| *R. stipulosus* R0154a | Ro07 | 95.45 | 1275 | 31 | 8 | 2 | 1262 | 892322 | 893583 | 0 | 1988 |
| *R. stipulosus* R0154b | Ro07 | 93.56 | 404 | 22 | 2 | 10 | 412 | 892351 | 892751 | 8.00e^-165^ | 583 |
| *R. stipulosus* R0154b | Ro07 | 93.18 | 616 | 25 | 7 | 409 | 1018 | 892990 | 893594 | 0 | 819 |
| *R. irenaeus* R2013a | Ro07 | 95.61 | 1254 | 28 | 9 | 6 | 1245 | 892352 | 893592 | 0 | 1955 |
| *R. irenaeus* R2013b | Ro07 | 93.43 | 396 | 22 | 2 | 20 | 414 | 892359 | 892751 | 4.00e^-160^ | 567 |
| *R. irenaeus* R2013b | Ro07 | 93.34 | 616 | 25 | 6 | 411 | 1021 | 892990 | 893594 | 0 | 835 |
| *R. pacificus* R2112a | Ro07 | 95.7 | 1302 | 31 | 8 | 1 | 1290 | 892324 | 893612 | 0 | 2042 |
| *R. pacificus* R2112b | Ro07 | 93.45 | 397 | 22 | 2 | 18 | 413 | 892358 | 892751 | 1.00e^-160^ | 569 |
| *R. pacificus* R2112b | Ro07 | 93.18 | 616 | 26 | 6 | 410 | 1020 | 892990 | 893594 | 0 | 827 |
| **22. Subsect. *Sozostyli* (Focke) Yü et Lu** |  |  |  |  |  |  |  |  |  |  |  |
| *R. playfarianus* R0110a | Ro07 | 95.54 | 493 | 6 | 4 | 12 | 496 | 892313 | 892797 | 0 | 795 |
| *R. playfarianus* R0110a | Ro07 | 96.33 | 762 | 21 | 2 | 490 | 1249 | 892827 | 893583 | 0 | 1283 |
| *R. playfarianus* R0110b | Ro07 | 93.14 | 408 | 23 | 3 | 6 | 412 | 892348 | 892751 | 4.00e^-160^ | 567 |
| *R. playfarianus* R0110b | Ro07 | 92.86 | 616 | 28 | 6 | 409 | 1019 | 892990 | 893594 | 0 | 811 |
| *R. bambusarum* R0145a | Ro07 | 95.38 | 455 | 6 | 3 | 4 | 450 | 892350 | 892797 | 0.00e^+00^ | 735 |
| *R. bambusarum* R0145a | Ro07 | 96.32 | 787 | 22 | 2 | 444 | 1228 | 892827 | 893608 | 0 | 1324 |
| *R. bambusarum* R0145b | Ro07 | 93.23 | 399 | 23 | 2 | 16 | 413 | 892356 | 892751 | 2.00e^-159^ | 565 |
| *R. bambusarum* R0145b | Ro07 | 92.89 | 647 | 30 | 6 | 410 | 1051 | 892990 | 893625 | 0 | 856 |
| *R. henryi* R0151a | Ro07 | 95.19 | 769 | 23 | 3 | 473 | 1239 | 892827 | 893583 | 0.00e^+00^ | 1229 |
| *R. henryi* R0151b | Ro07 | 93.58 | 405 | 22 | 2 | 3 | 406 | 892350 | 892751 | 2.00e^-165^ | 585 |
| *R. henryi* R0151b | Ro07 | 92.4 | 605 | 27 | 7 | 403 | 999 | 892990 | 893583 | 0 | 769 |
| *R. huangpingensis* R2010a | Ro07 | 95.35 | 1290 | 33 | 8 | 4 | 1280 | 892350 | 893625 | 0.00e^+00^ | 2002 |
| *R. huangpingensis* R2010b | Ro07 | 92.93 | 396 | 24 | 2 | 33 | 427 | 892359 | 892751 | 3.00e^-155^ | 551 |
| *R. huangpingensis* R2010b | Ro07 | 93.18 | 616 | 25 | 7 | 424 | 1033 | 892990 | 893594 | 0 | 819 |
| *R. swinhoei* R2015a | Ro07 | 95.33 | 1284 | 33 | 8 | 2 | 1271 | 892324 | 893594 | 0 | 1990 |
| *R. swinhoei* R2015b | Ro07 | 92.68 | 396 | 25 | 2 | 18 | 412 | 892359 | 892751 | 6.00e^-153^ | 543 |
| *R. swinhoei* R2015b | Ro07 | 93.02 | 616 | 26 | 7 | 409 | 1018 | 892990 | 893594 | 0 | 811 |
| *R. swinhoei* R2028a | Ro07 | 95.84 | 1299 | 28 | 7 | 1 | 1286 | 892323 | 893608 | 0 | 2076 |
| *R. swinhoei* R2028b | Ro07 | 92.84 | 405 | 25 | 2 | 7 | 410 | 892350 | 892751 | 3.00e^-158^ | 561 |
| *R. swinhoei* R2028b | Ro07 | 93.02 | 616 | 26 | 7 | 407 | 1016 | 892990 | 893594 | 0 | 811 |
| *R. swinhoei* R2129a | Ro07 | 95.52 | 1273 | 30 | 8 | 1 | 1259 | 892349 | 893608 | 0 | 1992 |
| *R. swinhoei* R2129b | Ro07 | 92.93 | 396 | 24 | 2 | 18 | 412 | 892359 | 892751 | 3.00e^-155^ | 551 |
| *R. swinhoei* R2129b | Ro07 | 92.87 | 617 | 27 | 7 | 409 | 1020 | 892990 | 893594 | 0 | 805 |
| *R. swinhoei* R2132a | Ro07 | 94.52 | 383 | 6 | 3 | 5 | 379 | 892350 | 892725 | 9.00e^-168^ | 593 |
| *R. swinhoei* R2132a | Ro07 | 95.94 | 838 | 22 | 5 | 391 | 1222 | 892764 | 893595 | 0 | 1312 |
| *R. swinhoei* R2132b | Ro07 | 93.09 | 405 | 24 | 2 | 7 | 410 | 892350 | 892751 | 1.00e^-160^ | 569 |
| *R. swinhoei* R2132b | Ro07 | 93.34 | 616 | 25 | 6 | 407 | 1017 | 892990 | 893594 | 0 | 835 |
| *R. caudifolius* R2001a | Ro07 | 94.67 | 1257 | 32 | 8 | 3 | 1246 | 892349 | 893583 | 0.00e^+00^ | 1863 |
| *R. caudifolius* R2001b | Ro07 | 93.24 | 370 | 21 | 2 | 6 | 374 | 892350 | 892716 | 6.00e^-147^ | 523 |
| *R. caudifolius* R2001b | Ro07 | 92.68 | 601 | 28 | 6 | 371 | 966 | 892994 | 893583 | 0 | 781 |
| *R. caudifolius* R2021a | Ro07 | 95.33 | 492 | 8 | 3 | 2 | 485 | 892313 | 892797 | 0 | 793 |
| *R. caudifolius* R2021a | Ro07 | 95.99 | 798 | 22 | 3 | 479 | 1271 | 892827 | 893619 | 0.00e^+00^ | 1231 |
| *R. caudifolius* R2021b | Ro07 | 93.24 | 370 | 21 | 2 | 6 | 374 | 892350 | 892716 | 6.00e^-147^ | 523 |
| *R. caudifolius* R2021b | Ro07 | 92.81 | 612 | 28 | 6 | 371 | 977 | 892994 | 893594 | 0 | 803 |
| *R. malifolius* R0109a | Ro07 | 96.03 | 1335 | 27 | 9 | 57 | 1380 | 892313 | 893632 | 0.00e^+00^ | 2117 |
| *R. malifolius* R0109b | Ro07 | 90.49 | 410 | 29 | 4 | 6 | 413 | 892350 | 892751 | 5.00e^-135^ | 484 |
| *R. malifolius* R0109b | Ro07 | 93.34 | 616 | 24 | 7 | 410 | 1019 | 892990 | 893594 | 0.00e^+00^ | 827 |
| *R. malifolius* R0147a | Ro07 | 96.1 | 1335 | 26 | 9 | 2 | 1325 | 892313 | 893632 | 0.00e^+00^ | 2125 |
| *R. malifolius* R0147b | Ro07 | 90.93 | 408 | 27 | 4 | 13 | 418 | 892352 | 892751 | 1.00e^-138^ | 496 |
| *R. malifolius* R0147b | Ro07 | 93.18 | 616 | 25 | 7 | 415 | 1024 | 892990 | 893594 | 0 | 819 |
| **Sect. *Dalibardastrum* (Focke) Yü et Lu** |  |  |  |  |  |  |  |  |  |  |  |
| *R. tsangorum* R2142a | Ro07 | 95.5 | 1334 | 32 | 10 | 58 | 1377 | 892313 | 893632 | 0 | 2066 |
| *R. tsangorum* R2142b | Ro07 | 93.09 | 405 | 24 | 2 | 8 | 411 | 892350 | 892751 | 1.00e^-160^ | 569 |
| *R. tsangorum* R2142b | Ro07 | 93.33 | 630 | 26 | 6 | 408 | 1032 | 892990 | 893608 | 0 | 854 |
| *R. amphidasys* R2115a | Ro07 | 95.58 | 1334 | 31 | 10 | 55 | 1374 | 892313 | 893632 | 0 | 2074 |
| *R. amphidasys* R2115b | Ro07 | 93.43 | 396 | 22 | 2 | 18 | 412 | 892359 | 892751 | 4.00e^-160^ | 567 |
| *R. amphidasys* R2115b | Ro07 | 93.03 | 617 | 27 | 6 | 409 | 1020 | 892990 | 893595 | 0 | 821 |
| **Sect. *Chamaebatus* Focke** |  |  |  |  |  |  |  |  |  |  |  |
| *R. calycinus* R2519a | Ro07 | 96.16 | 1277 | 25 | 7 | 3 | 1267 | 892328 | 893592 | 0.00e^+00^ | 2062 |
| *R. calycinus* R2519b | Ro07 | 95.07 | 406 | 16 | 2 | 6 | 410 | 892349 | 892751 | 2.00e^-180^ | 634 |
| *R. calycinus* R2519b | Ro07 | 86.64 | 614 | 61 | 10 | 407 | 1019 | 892990 | 893583 | 2.00e^-128^ | 462 |
| **Sect. *Cylactis* Focke** |  |  |  |  |  |  |  |  |  |  |  |
| *R. fockeanus* R2523a | Ro07 | 96.53 | 1268 | 21 | 6 | 7 | 1263 | 892328 | 893583 | 0.00e^+00^ | 2079 |
| *R. fockeanus* R2523b | Ro07 | 93.56 | 404 | 22 | 2 | 10 | 412 | 892351 | 892751 | 8.00e^-165^ | 583 |
| *R. fockeanus* R2523b | Ro07 | 93.53 | 618 | 25 | 5 | 409 | 1024 | 892990 | 893594 | 0 | 888 |
| *R. nyalamensis* R2534b | Ro07 | 94.7 | 396 | 17 | 2 | 22 | 416 | 892359 | 892751 | 5.00e^-172^ | 607 |
| *R. nyalamensis* R2534b | Ro07 | 94.07 | 607 | 21 | 5 | 413 | 1017 | 892990 | 893583 | 0 | 898 |
| *R. fragarioides* var. *pubescens* R2530a | Ro07 | 93.99 | 1265 | 32 | 9 | 3 | 1228 | 892324 | 893583 | 0.00e^+00^ | 1844 |
| *R. fragarioides* var. *pubescens* R2530b | Ro07 | 94.97 | 398 | 16 | 2 | 20 | 416 | 892357 | 892751 | 1.00e^-175^ | 618 |
| *R. fragarioides* var. *pubescens* R2530b | Ro07 | 93.85 | 618 | 22 | 6 | 413 | 1027 | 892990 | 893594 | 0 | 896 |
| **Sect. *Anoplobatus* Focke** |  |  |  |  |  |  |  |  |  |  |  |
| *R. odoratus* AF285994 | Ro07 | 82.86 | 245 | 10 | 3 | 3 | 219 | 891947 | 892187 | 3.00e^-45^ | 186 |
| *R. odoratus* AF285994 | Ro07 | 90.74 | 648 | 32 | 5 | 243 | 862 | 892205 | 892852 | 0 | 815 |
| *R. odoratus* AF285994 | Ro07 | 94.57 | 1104 | 44 | 5 | 867 | 1956 | 892911 | 894012 | 0.00e^+00^ | 1616 |

Note: Ro-*Rubus occidentalis*. * Jibran et al., 2018 [33].
