# Supplementary figures and images for: Allopolyploid origin in Rubus (Rosaceae) inferred from nuclear granule-bound starch synthase I (GBSSI) sequences
Source: BMC Plant Biol. 2019 Jul 10;19:303. doi: 10.1186/s12870-019-1915-7 (PMC6617891; doi:10.1186/s12870-019-1915-7)

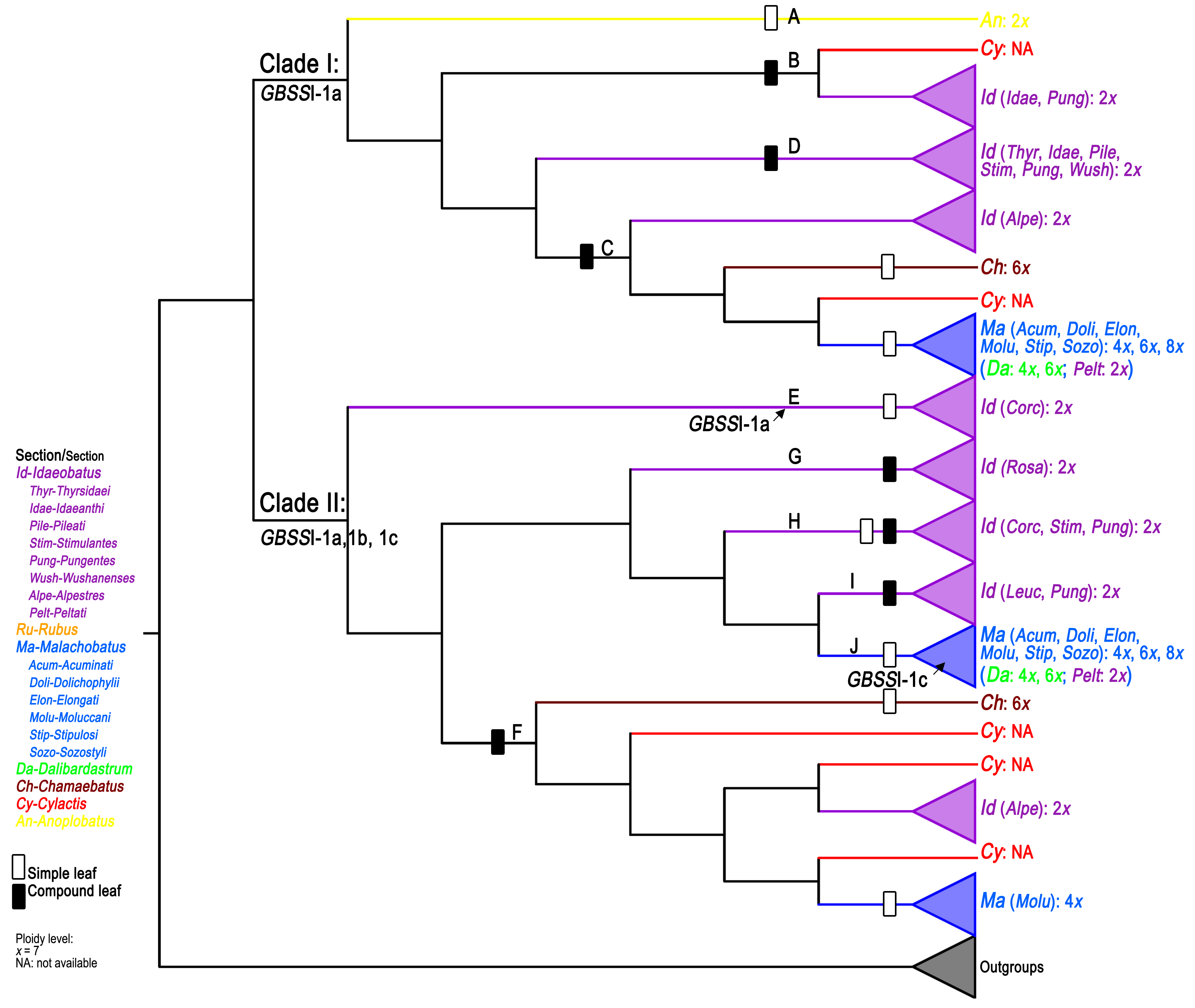

Supplement: Supplementary file 4 — The simplified ML tree corresponding to Figs. 3 and 4 in Rubus. (JPG 2328 kb) [file 12870_2019_1915_MOESM4_ESM.jpg]

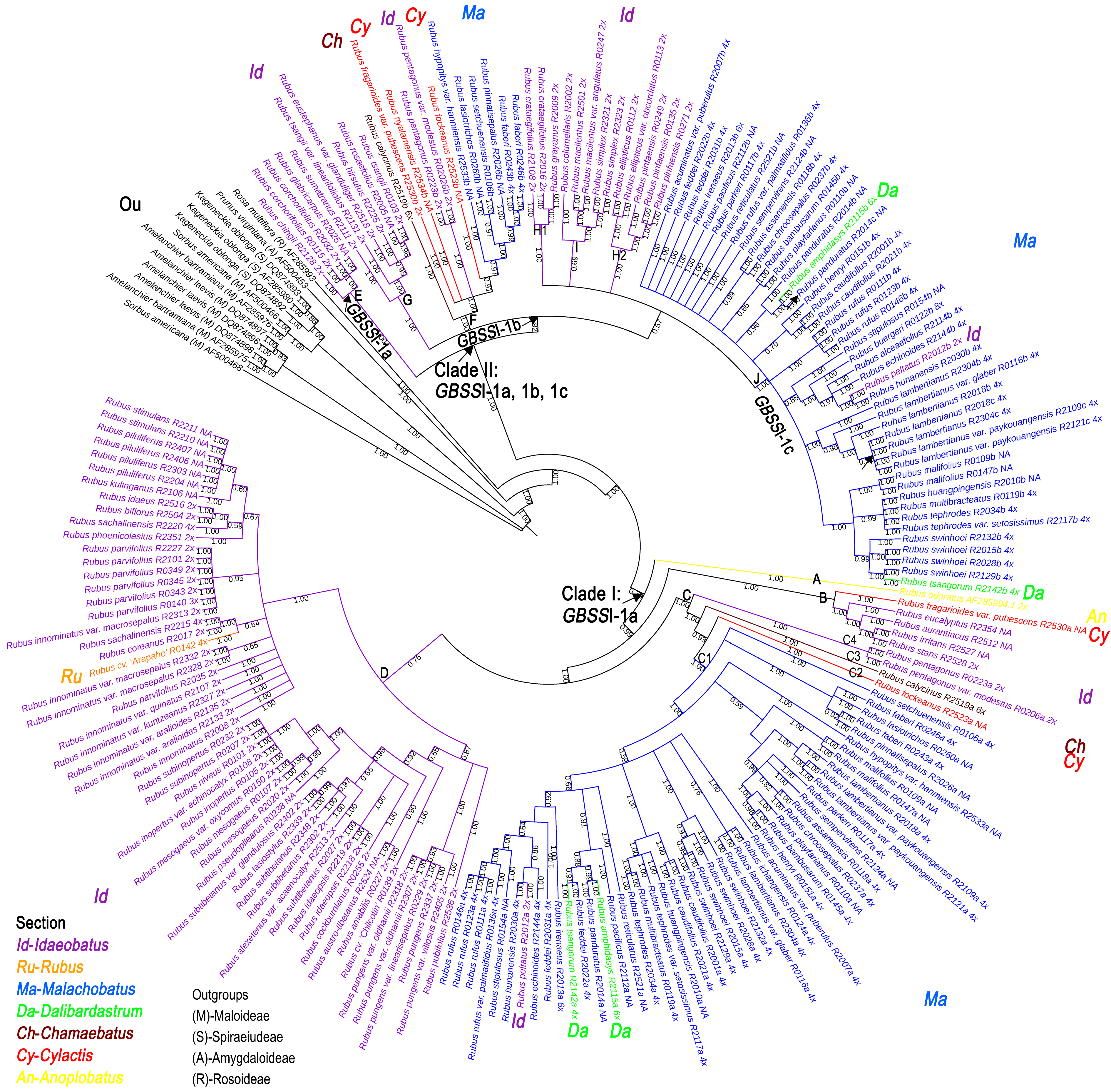

Supplement: Supplementary file 5 — Bayesian Inference (BI) tree inferred from the GBSSI-1 sequences of Rubus. Posterior probabilities >0.50 are shown below the branches. (JPG 7886 kb) [file 12870_2019_1915_MOESM5_ESM.jpg]
